# Unexpected regulatory functions of cyprinid Viperin on inflammation and metabolism

**DOI:** 10.1186/s12864-024-10566-x

**Published:** 2024-06-29

**Authors:** Lise Chaumont, Luc Jouneau, François Huetz, Doret R. van Muilekom, Mathilde Peruzzi, Claudine Raffy, Jérôme Le Hir, Jules Minke, Pierre Boudinot, Bertrand Collet

**Affiliations:** 1 Université Paris-Saclay, INRAE, UVSQ, VIM, 78350 Jouy-en-Josas, France; 2 Unit of Antibodies in Therapy and Pathology, UMR 1222 INSERM, Institut Pasteur, 75015 Paris, France; 3 VIRBAC S.A, 06510 Carros, France

**Keywords:** Viperin, RSAD2, Fathead minnow, *Pimephales promelas*, RNA-Seq, Interferon response, Inflammatory response, One carbon metabolism, Extracellular matrix, Cell adhesion

## Abstract

**Background:**

Viperin, also known as radical S-adenosyl-methionine domain containing protein 2 (RSAD2), is an interferon-inducible protein that is involved in the innate immune response against a wide array of viruses. In mammals, Viperin exerts its antiviral function through enzymatic conversion of cytidine triphosphate (CTP) into its antiviral analog ddhCTP as well as through interactions with host proteins involved in innate immune signaling and in metabolic pathways exploited by viruses during their life cycle. However, how Viperin modulates the antiviral response in fish remains largely unknown.

**Results:**

For this purpose, we developed a fathead minnow (*Pimephales promelas*) clonal cell line in which the unique *viperin* gene has been knocked out by CRISPR/Cas9 genome-editing. In order to decipher the contribution of fish Viperin to the antiviral response and its regulatory role beyond the scope of the innate immune response, we performed a comparative RNA-seq analysis of *viperin*^*−/−*^ and wildtype cell lines upon stimulation with recombinant fathead minnow type I interferon.

**Conclusions:**

Our results revealed that Viperin does not exert positive feedback on the canonical type I IFN but acts as a negative regulator of the inflammatory response by downregulating specific pro-inflammatory genes and upregulating repressors of the NF-κB pathway. It also appeared to play a role in regulating metabolic processes, including one carbon metabolism, bone formation, extracellular matrix organization and cell adhesion.

**Supplementary Information:**

The online version contains supplementary material available at 10.1186/s12864-024-10566-x.

## Background

The host innate immune system is the first line of defense against viral infections. Innate antiviral defenses are primarily based on type I interferons (IFNs), which are cytokines secreted upon the recognition of viruses. Type I IFNs bind to cell surface class II cytokine receptors and elicit the expression of hundreds of IFN-stimulated genes (*ISGs*) through the JAK-STAT signaling cascade [1]. *ISGs* are engaged in diverse functions within the cell, and include virus sensors, receptors, transcription factors, signaling adaptors involved in upstream molecular signaling cascades as well as other cytokines, which enhance the IFN response. Other *ISGs* encode antiviral effectors, which directly target specific viral components or modulate pathways and/or functions required during the virus life cycle. Altogether, *ISG* products participate in mounting an antiviral state refractory to viral infection, replication and propagation [2].

The radical S-adenosyl-methionine (SAM) domain-containing protein 2 (RSAD2), also known as virus inhibitory protein endoplasmic reticulum-associated, IFN-inducible (Viperin), ranks among the most highly induced *ISGs* upon stimulation with IFNs, dsRNA, viral infections; it is also induced upon lipopolysaccharide stimulation and bacterial infections [3–5]. The *viperin* transcript was initially identified by differential display analysis in primary human fibroblasts infected with human cytomegalovirus (HCMV) [6], later described in rainbow trout (*Oncorhynchus mykiss*) leukocytes infected with viral hemorrhagic septicemia virus (VHSV) [7] and further characterized from primary human macrophages treated with IFN-γ [8]. Structurally, Viperin is composed of three distinct domains: an N-terminal domain that greatly varies in length among vertebrates [5, 9] and contains an amphipathic alpha helix mediating its localization to the cytosolic face of the endoplasmic reticulum and lipid droplets [10, 11], a conserved central domain bearing the canonical CX_3_CX_2_C motif, which is characteristic of the radical SAM superfamily and coordinates the binding to a [Fe_4_S_4_] cluster required for its enzymatic activity, and a conserved C-terminal domain of unknown function [7, 12]. Importantly, *viperin* genes are widely conserved among both vertebrates and invertebrates [4, 13]. *viperin*-like genes have also recently been identified across all kingdoms of life, including fungi, bacteria, and archaea, hinting at an ancient mechanism possibly connected to antiviral defense [12, 14, 15].

In mammals, Viperin inhibits a broad spectrum of DNA and RNA viruses, [16], although its capacity to limit viral replication may drastically differ from one virus to another [17, 18]. Viperin exerts its antiviral action through different mechanisms, involving its enzymatic activity and/or protein–protein interactions [16]. It was reported early on that Viperin often had to be catalytically active to exert its antiviral action [4, 19], but its substrate remained elusive for many years [20]. Recent biochemical studies have demonstrated that it converts cytidine triphosphate (CTP) to its analog 3′-deoxy-3′,4′-didehydro-cytidine triphosphate (ddhCTP) through a SAM-dependent radical mechanism [21]. ddhCTP was shown to inhibit the replication of some RNA viruses by acting as a natural chain terminator for RNA-dependent RNA polymerases, although this mechanism has recently been challenged [22]. Besides its radical SAM enzymatic activity, Viperin also interacts with a wide range of viral and cellular proteins. It was reported to bind viral proteins and to promote their degradation through the proteasomal pathway [18, 23]. Furthermore, Viperin interacts with cellular mediators involved in innate immune signaling, including ssRNA-sensing TLR7 pathway, unmethylated CpG DNA-sensing TLR9 pathways [24, 25] and cytosolic dsDNA cGAS-STING pathway [26], thereby enhancing the IFN response. Viperin was also reported to be in the oxidation of methionine residues in DNA and RNA helicases, including the cytosolic viral RNA sensor RIG-I, which increases its stability leading to enhanced expression of IFN-β in Mouse Embryonic Fibroblasts (MEFs) [27]. Furthermore, a growing body of evidence suggests that Viperin modulates metabolic pathways exploited during the viral life cycle, including cholesterol biosynthesis [28, 29] and secretion of soluble proteins [10, 30]. A few additional studies point to a role of Viperin in the regulation of metabolic processes under non-infectious conditions, including bone and cartilage formation [31, 32], reduction of fatty acid β-oxidation [33, 34] and regulation of the mitochondrial metabolism [35]. Nonetheless, how Viperin can have such broad cellular functions is currently still unclear.

In fish, orthologs of the mammalian *viperin* gene have been identified in many species [7, 9, 36–38]. Recently, Wang et al. have cloned the *viperin* gene from fathead minnow (*Pimephales promelas*) FHM cells [39]. Intriguingly, they have also identified a splicing variant lacking exon 5, that is expressed upon Spring viremia of carp virus (SVCV) infection but not poly(I:C) stimulation. Several studies have reported that fish Viperins, including Viperin from fathead minnow, retain antiviral properties [9, 37–39]. Furthermore, although studies on fish Viperin have not investigated the underlying molecular mechanisms as thoroughly as in mammals, they still provide evidence that fish Viperin is able to modulate the expression of genes involved in IFN and inflammatory response following overexpression in fish cell lines [9, 38–40], stabilize RIG-I by increasing its half-life [40], interact with viral proteins to promote their degradation via the proteasomal and/or autophagosome pathways [36] and modulate cholesterol metabolism [41]. Importantly, most studies were performed using overexpression approaches and to the best of our knowledge, no knockout in vitro models have been developed so far using fish cell lines.

In order to better understand the contribution of fish Viperin to the antiviral response and its regulatory role beyond the scope of the innate immune response, we developed a clonal epithelial-like cyprinid cell line in which the *viperin* gene has been knocked out by CRISPR/Cas9 genome-editing. This cell line derives from the fathead minnow Epithelioma papulosum cyprini (EPC) cell line, which is widely used in diagnostic and research. To achieve a global overview of the transcriptional response between the knockout and the wildtype cell lines, we performed a comparative RNA-seq analysis of the whole transcriptome of the two cell lines with or without a 24 h-long stimulation with recombinant fathead minnow type I IFN. Our transcriptomic analysis indicates that Viperin is not involved in the regulation of the canonical type I IFN in this model but acts as a negative regulator of specific inflammatory pathways. In addition, our study sheds light on other metabolic functions in which Viperin may play a role even under non-pathological conditions, including extracellular matrix organization, cell adhesion, bone formation and one carbon metabolism.

## Methods

### Cell lines, culture conditions and viruses

The *Epithelioma papulosum cyprini* (EPC) cell line (ATCC CRL-2872, *Pimephales promelas*), was grown in Leibovitz’s L-15 medium (Gibco) supplemented with 10% fetal bovine serum (FBS, Eurobio) and penicillin (100 U/mL)-streptomycin (100 μg/mL) (BioValley). The EPC-EC cell line (described below) and its derivatives were grown in L-15 medium supplemented with 10% FBS, penicillin (100 U/mL)-streptomycin (100 μg/mL), 500 μg/mL G418 (Invivogen), 30 μg/mL hygromycin B Gold (Invivogen). The Chinook salmon (*Oncorhynchus tshawytscha)* embryo (CHSE-214) cell line was maintained in Glasgow’s modified Eagle’s medium (GMEM) containing 25 mM HEPES (Biosera) supplemented with 10% FBS, 2 mM L-glutamine (Eurobio), and penicillin (100 U/mL)-streptomycin (100 μg/mL). All cell lines were maintained at 20 °C without CO_2._

Recombinant viral hemorrhagic septicemia virus expressing the tdTomato red fluorescent protein (rVHSV-Tomato) was a kind gift from Dr. Stéphane Biacchesi (Université Paris-Saclay, INRAE, UVSQ, VIM, Jouy-en-Josas, France) [42]. rVHSV-Tomato was propagated in EPC cells (multiplicity of infection (MOI) of 1); briefly, the virus was adsorbed onto the cells for 1 h at 14 °C with regular gentle shaking; L-15 supplemented with 2% heat-inactivated FBS was added afterwards and the supernatants were collected at 5 days post-infection, 0.2 µm-filtered, aliquoted and stored at -80 °C. Infectious pancreatic necrosis virus (IPNV), isolate 31.75 [43], was propagated in CHSE-214 (MOI 0.001) at 14 °C in GMEM supplemented with 2% FBS, as described above for rVHSV-Tomato. The supernatants were collected at 3–4 days post-infection, 0.2 µm-filtered, diluted 1:5 (v/v) in TEN buffer (10 mM Tris, 1 mM EDTA, 150 mM NaCl, pH 7.1) and mixed again 1:1 (v/v) in glycerol 100%, aliquoted and stored at -20 °C. rVHSV-Tomato and IPNV31.75 titers were determined by plaque assay.

### Development and validation of a viperin−/− cell line

The EPC cell line was genetically engineered to overexpress a monomeric, cytosolic form of EGFP (mEGFP) and the nuclear nCas9n using the same method used to develop the CHSE-EC cell line [44]. Briefly, the EPC cell line was engineered using the plasmid pcDNA3.1-Hyg-nCas9n (Addgene #217,487); single cells were individualized by flow cytometry sorting (BD FACSAria™ II Cell Sorter, INEM, Paris, France) and after propagation, clones expressing high levels of *nCas9n* transcripts were selected by RT-qPCR, as previously described [44]. The resulting cell line was engineered a second time using the plasmid pmEGFP-N1 (Addgene #217,486); single mEGFP-positive cells were isolated by FACS (BD FACSAria™ III Cell Sorter, INRAE, Jouy-en-Josas, France) to generate a clonal cell line, named EPC-EC.

The EPC-EC cell line was used to develop a *viperin*^*−/−*^ cell line. Two single guide RNAs (sgRNAs) were designed within the first exon of the *viperin* gene (LOC120476724), using CRISPOR v5.01 web tool (Table 1) [45]. To ensure the specificity of the sgRNAs, care was taken that no off-target genes with more than 3 mismatches in the first 12 bp adjacent to the PAM (most likely off-targets) were identified in the fathead minnow genome (EPA_FHM_2.0, NCBI RefSeq assembly GCF_016745375.1). A sgRNA targeting the *mEGFP* gene was also used as previously designed [44].

**Table 1:** Primers used in this study

Primer name	Sequence (5′3’)	Source or reference	Specificities
Plasmid constructs			
BFP-F	CTGCTGCT***GGCTAGC***TCTAGA**CTCGAG**ATGAGCGAGCTGATTAAGGAGA	pCite-P-BFP	***NheI*** XbaI **XhoI**
BFP-R	CAGCAGCAG***AAGCTT***GGTACC**CTGCAG***GGATCC***GATATC**GTGCCCCAGTTTGCTAGG	pCite-P-BFP	***HindIII*** KpnI **PstI** *BamHI* **EcoRV**
PpViperin-R0-F	ccaagttggttttgcaagaATGT	JNCE01171228 XM_039667881.1	
PpViperin-R0-R	attgagaaaggTCACCACTCC	JNCE01171228 XM_039667881.1	
PpViperin-P2A-F	GGAAGCGGAGCTACTAACTTCAGCCTGCTGAAGCAGGCTGGAGACGTGGAGGAGAACCCTGGACCTATGTTGATGCCATTGTGTTTCAAGG	JNCE01171228 XM_039667881.1	P2A
PpViperin-HindIII-R	CTGCTGCTG***AAGCTT***TCACCACTCCAGTTTCATATCTTCC	JNCE01171228 XM_039667881.1	**HindIII**
sgRNA			
sgRNA-mEGFP-S	**TCCTAATACGACTCACTATA**GGCGAGGGCGATGCCACCTA*GTTTTAGAGCTAGAAATAGCAAGTTAAAATAAGGCTAGTCCGTTATCAACTTGAAAAAGTGGCACCGAGTCGGTGCTTTT*	[44]	**T7 promoter** sgRNA target *Scaffold*
sgRNA-mEGFP-AS	*AAAAGCACCGACTCGGTGCCACTTTTTCAAGTTGATAACGGACTAGCCTTATTTTAACTTGCTATTTCTAGCTCTAAAAC*TAGGTGGCATCGCCCTCGCC**TATAGTGAGTCGTATTAGGA**	[44]	**T7 promoter** sgRNA target *Scaffold*
sgRNA-Vip1-S	**TCCTAATACGACTCACTATA**CGAACGAGGTCTTCGCAGTG*GTTTTAGAGCTAGAAATAGCAAGTTAAAATAAGGCTAGTCCGTTATCAACTTGAAAAAGTGGCACCGAGTCGGTGCTTTT*	XM_039667881.1 Coding exon 1	**T7 promoter** sgRNA target *Scaffold*
sgRNA-Vip1-AS	*AAAAGCACCGACTCGGTGCCACTTTTTCAAGTTGATAACGGACTAGCCTTATTTTAACTTGCTATTTCTAGCTCTAAAAC*CACTGCGAAGACCTCGTTCG**TATAGTGAGTCGTATTAGGA**	XM_039667881.1 Coding exon 1	**T7 promoter** sgRNA target *Scaffold*
sgRNA-Vip2-S	**TCCTAATACGACTCACTATA**TGGAGTGGTCACCTGTGCGC*GTTTTAGAGCTAGAAATAGCAAGTTAAAATAAGGCTAGTCCGTTATCAACTTGAAAAAGTGGCACCGAGTCGGTGCTTTT*	XM_039667881.1 Coding exon 1	**T7 promoter** sgRNA target *Scaffold*
sgRNA-Vip2-AS	*AAAAGCACCGACTCGGTGCCACTTTTTCAAGTTGATAACGGACTAGCCTTATTTTAACTTGCTATTTCTAGCTCTAAAAC*GCGCACAGGTGACCACTCCA**TATAGTGAGTCGTATTAGGA**	XM_039667881.1 Coding exon 1	**T7 promoter** sgRNA target *Scaffold*
Genotyping			
mEGFP-gen-F	GGCACCAAAATCAACGGGAC	pmEGFP-N1	
mEGFP-gen-R	GCCGTCGTCCTTGAAGAAGA	pmEGFP-N1	
PpViperin-gen-F	CACACTTCACCACATCAAACCA	LOC120476724	
PpViperin-gen-R	GGTGACATGTTAGATTACCTGCTTC	LOC120476724	

The sgRNAs were synthesized using the T7 RiboMAX™ Express Large Scale RNA Production System kit (Promega) according to the manufacturers’ instructions using 0.5 µg of each primer that spontaneously annealed as the dsDNA template. The RNA synthesis mix was then incubated with 1 µL of RQ1 DNase (Promega) for 1 h at 37 °C and purified using TRIzol™ reagent (Invitrogen), according the manufacturers’ instructions. The sgRNAs were resuspended in RNase- and DNase-free water and quantified using a Nanodrop spectrophotometer. The purity of the sgRNAs was checked on a 2% agarose-EtBr gel before or after a 30 min treatment with RNase A (Qiagen) at room temperature. The ability of each sgRNA to cut the target sequence was confirmed by in vitro efficiency assay. Briefly, genomic DNA was extracted from ~ 3 × 10^6^ EPC-EC cells using NucleoSpin Tissue Mini kit (Macherey–Nagel), according to the manufacturer’s instructions. Genomic DNA segments containing the targeted sites were amplified by PCR using GoTaq® G2 Flexi DNA Polymerase (Promega) with the primers mEGFP-gen-F/mEGFP-gen-R and PpViperin-gen-F/PpViperin-gen-R (Table 1). The PCR cycling program was performed in a thermal cycler (Eppendorf) and was as follows: 94 °C for 3 min then 35 cycles of 94 °C for 15 s, 59 °C for 15 s, 72 °C for 40 s, and a final extension of 72 °C for 5 min. The PCR products were purified using NucleoSpin Gel and PCR Clean-up Mini kit (Macherey–Nagel). Each sgRNA was mixed with recombinant TrueCut™ Cas9 Protein v2 (Invitrogen) at a 1:1 molar ratio (0.2 µg sgRNA and 1 µg rCas9 ie. 6.1 pmol each in 12 µL of resuspension buffer R (Neon™ Transfection System kit, Invitrogen)) and incubated at room temperature for 15 min. Each sgRNA/Cas9 complex was mixed with the purified PCR product at a 5:1 molar ratio (ie. 6.1 pmol sgRNA/Cas9 and 1.22 pmol PCR product) and incubated at room temperature overnight. The Cas9 enzyme was heat-inactivated at 80 °C for 20 min and double-strand break of the PCR products was confirmed on a 1.5% agarose-EtBr gel.

To generate *viperin*^*−/−*^ cells, each sgRNA (sgRNA-mEGFP, sgRNA-Vip1, sgRNA-Vip2) was mixed with recombinant TrueCut™ Cas9 Protein v2 at a 1:1 molar ratio (0.2 µg sgRNA and 1 µg rCas9 ie. 6.1 pmol each in 2 µL) and incubated at room temperature for 20 min. The sgRNA-mEGFP/Cas9 complex was mixed with pooled sgRNA-Vip1/Cas9 + sgRNA-Vip2/Cas9 complexes at a 2:1 volume ratio in resuspension buffer R (Neon™ Transfection System kit, Invitrogen) (ie. 1µL of sgRNA-mEGFP/Cas9 and 0.5 µL of each sgRNA-Vip/Cas9 complex in a final volume of 5 µL). The mix was transfected into EPC-EC cells using the Neon™ Transfection System (Invitrogen). EPC-EC cells were prepared as described in the “transfections” section and 5 µL of cell suspension at 2 × 10^7^ cells/mL was mixed with 5 µL of sgRNA/Cas9 complex (*i.e.* 1 × 10^5^ cells, 6.1 pmol of Cas9 and 6.1 pmol of sgRNA per 10 µL of transfection reaction). The cells were transfected using the same conditions established for plasmids, as described in the “transfections” section. All transfected cells (~ 5 × 10^5^ cells) were mixed in 5 mL L-15 supplemented with 10% FBS and penicillin (100 U/mL)-streptomycin (100 μg/mL) in a 25 cm^2^ flask (Sarstedt) and incubated at 20 °C. The next day, cells were washed with PBS, fresh medium (L-15 medium supplemented with 10% FBS, penicillin (100 U/mL)-streptomycin (100 μg/mL), 500 μg/mL G418, 30 μg/mL hygromycin B Gold) was added into each flask and cells were incubated at 20 °C for 4 weeks.

Once the cell population reached confluency, the transfected cells were passaged (surface ratio 1:4), and ~ 3 × 10^6^ cells were used for genomic DNA extraction using NucleoSpin Tissue Mini kit (Macherey–Nagel), according to the manufacturer’s instructions. Genomic DNA regions containing the sgRNA-targeted sequences were amplified by PCR using genotyping primers mEGFP-gen-F/mEGFP-gen-R and PpViperin-gen-F/PpViperin-gen-R, as described above. The PCR products were purified using NucleoSpin Gel and PCR Clean-up Mini kit (Macherey–Nagel) and directly sequenced using the same amplification primers. Sequences were analyzed using Synthego ICE analysis tool v3 (Synthego) [46] to assess the percentage of mutated cells in the transfected cell population (bulk). ICE analysis confirmed efficient genome editing for *mEGFP* and *viperin* in the bulk transfected with sgRNA-mEGFP and sgRNA-Vip1 + 2. This bulk was further used for isolation of *viperin*^*−/−*^ clones by FACS. For this purpose, cells were detached by trypsin–EDTA action and mEGFP-deficient single cells at a density of ~ 4 × 10^6^ cells/mL were individualized by FACS (BD FACSAria™ Fusion Flow Cytometer, Institut Pasteur, Paris, France) using a 100 µm nozzle at the lowest pressure (1 out of a scale of 11) into a 96-well plate (Sarstedt) in L-15 supplemented with 10% FBS, penicillin (200 U/mL)-streptomycin (200 μg/mL), 500 μg/mL G418 and 30 μg/mL hygromycin B Gold. Three months later, 16 clones were sub-cultured and propagated in 25 cm2 flasks and their genotype was characterized as described above. Two clones, EPC-EC-Vip-C7 and EPC-EC-Vip-C11, were kept for further knockout validation. Because both clones presented heterozygous mutations at the cutsites targeted by the two sgRNA used, the previously obtained genotyping PCR products were cloned into the pCR4-TOPO TA vector using TOPO™ TA Cloning™ kit (Invitrogen), according to the manufacturer’s instructions, and sent to sequencing. The sequencing results showed that both clones presented heterozygous mutations at both sgRNA targeted sites: a 1-nt deletion (152delC) or 2-nt deletion (152_153delAC) at sgRNA-Vip2 target site and a partial 1-nt insertion (230_231insT) at sgRNA-Vip1 target site, resulting in frameshifts and the appearance of a premature stop codons (D51fsX53 or G52fsX96).

The disruption of *viperin* was also validated by western blot. For this purpose, EPC-EC-Vip-C7 and EPC-EC-Vip-C11 clones and the WT EPC-EC cell line were seeded into 25 cm2 flasks at a density of 6.5 × 10^6^ cells/well in L-15 supplemented with 2% FBS and penicillin (100 U/mL)-streptomycin (100 μg/mL) and incubated at 20 °C overnight. The next day, the cells were stimulated with poly(I:C) (Sigma-Aldrich) diluted in L-15 supplemented with 10% FBS and penicillin (100 U/mL)-streptomycin (100 μg/mL) at a final concentration of 500 µg/mL or left untreated and incubated at 20 °C. At 24, 48 and 72 h post-stimulation, medium was removed, cells were washed once with ice-cold DPBS, scraped in 1 mL ice-cold DPBS supplemented with 2.5 mM EDTA and centrifuged at 1500 g at 4 °C for 5 min. The cell pellets were drained, resuspended in 100 µL NP-40 lysis buffer (50 mM Tris–HCl pH 7.4, 150 mM NaCl, 2 mM EDTA, 0.5% NP40, 1 mM DTT, 10% glycerol, cOmplete™ protease inhibitor (Merck)) and lysed for 45 min at 4 °C under gentle shaking. The cell lysates were clarified by centrifugation at 5000 g at 4 °C for 5 min and stored at -80 °C until use.

Aliquots of 60 μL of cell lysates were mixed with 30 μL Laemmli buffer 3X (45 mM Tris, 345 mM glycine, 38% glycerol, 4.8% SDS, 20% β-mercaptoethanol, 0.04% bromophenol blue) and incubated at 100 °C for 5 min. A volume of 8 µL of cell lysates was loaded onto 12% polyacrylamide gels and protein samples were separated by electrophoresis in Tris–glycine buffer (25 mM Tris, 192 mM glycine, pH 8,3). Proteins were then transferred onto a nitrocellulose membrane (BioRad) using the mixed molecular weight program from the Trans-Blot® Turbo™ Transfer System (BioRad). The blots were blocked with 5% non-fat milk in TBST (10 mM Tris pH 7.4, 150 mM NaCl, 0,1% Tween 20) for 1 h at room temperature and then incubated with rabbit polyclonal anti-Viperin antibody (PA5-42,231, Invitrogen) (1:500, TBST + 5% non-fat milk) overnight at 4 °C. Care was taken to use an antibody raised against an immunogenic polypeptide (Tyr301-Tyr350, human RSAD2) that shares 92% identity with the corresponding sequence on fathead minnow Viperin (XP_039523815.1). The blots were washed 5 times in TBST, incubated with horseradish peroxidase (HRP)-conjugated anti-mouse or anti-rabbit (1:4000) secondary antibodies (SeraCare), washed 4 times in TBST and once in PBS. Western blots were developed using Clarity™ Western ECL substrate (BioRad) and detected using ChemiDoc Touch Imaging System (BioRad). After the first detection, the membrane was washed twice with TBST, saturated with TBST-5% non-fat milk for 1 h and re-probed with mouse monoclonal anti-α-tubulin antibody (T9026, Sigma Aldrich) (1:3000, TBST + 5% non-fat milk) for 2.5 h-3 h and developed as described above. Densitometric analysis of the blots was performed using Image Lab software (v 6.1.0, BioRad).

### Plasmid constructions

Fathead minnow *viperin* open reading frame (ORF) sequence was identified in silico using NCBI Reference EPA_FHM_2.0 Primary Assembly and predicted transcript XM_039667881.1.

Total RNA from 4 × 10^6^ EPC-EC cells infected with IPNV31.75 (MOI 0.001) at 72 h post-infection in quadruplicates was extracted using the QiaShredder and RNeasy mini kits (Qiagen) according to the manufacturer’s instructions. RNA (4 µg) was used as template for reverse transcription and generation of cDNA using the iScript™ Advanced cDNA Synthesis Kit for RT-qPCR (BioRad) and the synthesis was performed in a thermal cycler (Eppendorf) as recommended by the manufacturer. cDNA was diluted to 1:20 in DNase- and RNase-free water. Diluted cDNA from rVHSV-Tomato infected cells (*n* = 4) were pooled and used as template to amplify the *viperin* ORF sequence. Nested PCR amplifications were performed using Q5 2X High-Fidelity mastermix (New England Biolabs) and 2 sets of specific primers according to the manufacturer’s instructions: PpViperin-R0-F/PpViperin-R0-R were used for the first PCR round while PpViperin-P2A-F and PpViperin-HindIII-R were used for the second PCR round (Table 1). The PCR cycling programs were performed in a thermal cycler (Eppendorf) and were as follows: 98 °C for 30 s followed by 35 cycles of 98 °C for 10 s, 65 °C for 10 s, 72 °C for 90 s (1st round) or 60 s (2nd round), and a final extension of 72 °C for 2 min. PCR products were purified with the NucleoSpin Gel and PCR Clean-up Mini kit (Macherey–Nagel), quantified using a Nanodrop spectrophotometer, digested with HindIII enzymes (Thermofisher), cloned into HindIII/EcoRV-digested pcDNA3.1-Hyg-BFP vector (Addgene #214363) using T4 DNA ligase (New England Biolabs) according to the manufacturer’s instructions and fully sequenced. The pcDNA3.1-Hyg-BFP vector was initially obtained by amplifying the BFP gene from pCite-P-BFP [47] using BFP-F/BFP-R primers (Table 1) and subcloning it into the plasmid backbone of pcDNA3.1-Hyg-mEGFP (Addgene #191847) by XhoI/HindIII digestion. The pCite-PBFP plasmid was a kind gift from Dr. Hortense Decool (Université Paris-Saclay, INRAE, UVSQ, VIM, France). The resulting plasmid was named pcDNA3.1-Hyg-BFP-P2A-PpViperin (Addgene #217481). All plasmids were produced in Stellar™ Competent Cells (Takara) and were purified using NucleoBond Xtra Maxi EF (Macherey–Nagel) according to the manufacturer's instructions.

### Transfections

Transfections were performed by electroporation using the Neon™ Transfection System (Invitrogen) as described previously [44]. Briefly, EPC cells were washed in DPBS (Sigma-Aldrich), detached by trypsin–EDTA action, resuspended in L-15 supplemented with 10% FBS and penicillin (100 U/mL)-streptomycin (100 μg/mL) and centrifuged at 400 g for 5 min. The cell pellet was drained, resuspended in L-15 without Phenol Red (Gibco), centrifuged at 13 000 g for 30 s, and resuspended again in L-15 without Phenol Red. The cell concentration was adjusted to 2 × 10^7^ cells/mL. The cell suspension was mixed either with pcDNA3.1-Hyg-BFP or pcDNA3.1-Hyg-BFP-P2A-PpViperin at a final concentration of 5 µg per 1 × 10^6^ cells per 100 µL of transfection reaction. The fluorescent vector pcDNA3.1-Hyg-RFP-KDEL (Addgene #138,660; 2 µg/1 × 10^6^ cells/100 µL of transfection reaction) was added to check transfection efficiency between each condition. Transfections were carried out in an electroporator MPK5000 (Neon™ Transfection System, Invitrogen) using a 100 μL transfection kit (Neon™ Transfection System, Invitrogen) set to two pulses for 20 ms at 1400 V, as previously established for EPC cells [48]. All transfected cells (~ 3 × 10^6^ cells) were mixed in L-15 supplemented with 10% FBS and penicillin (100 U/mL)-streptomycin (100 μg/mL), incubated at 20 °C. At 24 h post-transfection, medium was removed, cells were washed once with ice-cold DPBS and directly lysed in Laemmli buffer (45 mM Tris, 345 mM glycine, 38% glycerol, 4.8% SDS, 20% β-mercaptoethanol, 0.04% bromophenol blue) for 45 min at 4 °C. Cell lysates were collected, incubated at 100 °C for 5 min and stored at -80 °C until use for western blot analysis.

### Real-time quantitative PCR

EPC-EC cells were seeded in 6-well plates to a final density of 2.5 × 10^6^ cells/well in L-15 supplemented with 2% heat-inactivated FBS and penicillin (100 U/mL)-streptomycin (100 μg/mL) and incubated overnight at 20 °C. The next day, cells were either infected with rVHSV-Tomato (MOI 0.05) or IPNV31.75 (MOI 0.001) at 14 °C for 24, 48 or 72hpi (n = 4 for each time point), stimulated with recombinant *Pimephales promelas* type I IFN supernatant diluted to 1:10 in L-15 supplemented with 2% FBS and penicillin (100 U/mL)-streptomycin (100 μg/mL) for 24 h or left untreated (n = 3 for each condition). Recombinant *Pimephales promelas* type I IFNφ1 supernatant was a kind gift from Dr. Stéphane Biacchesi (Université Paris-Saclay, INRAE, UVSQ, VIM, Jouy-en-Josas, France) and was produced as previously described [49].

Total RNA was extracted from cells in individual P6 wells in triplicates or quadruplicates using QiaShredder and RNeasy mini kits (Qiagen) in accordance with the manufacturer’s instructions. Quality control of the samples was determined using a Nanodrop spectrophotometer. The cDNA was generated from 4 µg (VHSV experiment) or 3 µg (IFN experiment) of total RNA using the iScript™ Advanced cDNA Synthesis Kit for RT-qPCR (BioRad) and the synthesis was performed in a thermal cycler (Eppendorf) as recommended by the manufacturer. cDNA was diluted in DNase- and RNase-free water to reach a final concentration of 10 ng/µL and stored at -20 °C until use. “No RT” control reactions were made by omitting the reverse transcriptase.

The cDNA was mixed with iTaq™ Universal SYBR® Green SupermixTB (Biorad) along with forward and reverse primers (Table 2) at a final concentration of 300 nM each in Twin.tec® real-time PCR plates (Eppendorf). Amplification was performed using a Realplex^2^ Mastercycler (Eppendorf) using the following cycling program: initial denaturation at 95 °C for 3 min followed by 40 cycles of 10 s at 95 °C and 30 s at 60 °C. For each biological replicate, mean Ct values of target genes were calculated based on technical duplicate reactions and then normalized using Ct values of a housekeeping gene (*Ppactin*). The relative expression of each target gene was expressed as 2^−∆Ct^, which was then used to calculate their respective fold change in comparison to non-stimulated cells. For each set of primers, the efficiency was calculated by linear regression obtained by using five-fold serial dilutions of a pool of cDNA and the qPCR products were validated by sequencing.

**Table 2:** qPCR primers used in this study

Name	Sequence 5’-3’	Target name	Target accession number	Size	Reference
Ppactin-ex-F	TGACGCAGATCATGTTCGAGA	Beta actin	XM_039687266.1 XM_039652364.1	255 bp	This study
Ppactin-ex-R	CCGTGGTGGTGAAGCTGTAA				
Ppviperin-ex-F	AGAGGCAAAGCGAGGGTTAC	Radical SAM domain containing 2	XM_039667881.1	214 bp	This study
Ppviperin-ex-R	GTCCAAGTAGTCACCGTATTTCTG				
Ppmx1-ex-F	CCAGGGGTAGTGGAATTGTTACA	Interferon-induced GTP-binding protein Mx	XM_039657463.1	161 bp	This study
Ppmx1-ex-R	CTCATCCTGGGCTTCACGAA				
Pppkr-ex-F	ACAGAGACCTGAAGCCTCCAA	Eukaryotic translation initiation factor 2-alpha kinase 2	XM_039649056.1	173 bp	This study
Pppkr-ex-R	GGATGTTTGAGTCGCTTGCTC				
Ppstat2-ex-F	TCAAAGTAGAGGTGATGGAGCA	Signal transducer and activator of transcription 2	XM_039689152.1	206 bp	This study
Ppstat2-ex-R	AGCACCATCCAACATAGCCG				

### RNA-Seq analysis

#### Cell stimulation and RNA extraction

WT EPC-EC cells and *viperin*^*−/−*^ EPC-EC-Vip-C7 cells were seeded, stimulated with recombinant *Pimephales promelas* type I IFNφ1 supernatant or left untreated, and total RNA was extracted and quantified, as described in the “RT-qPCR” section. To remove any contaminating DNA, 3 µg of total RNA from each sample were DNase-treated, using Turbo DNA-free™ kit (Invitrogen), according to the manufacturer’s instructions.

#### Illumina sequencing and mapping of reads

Sequencing of RNA samples was performed at I2BC sequencing platform (Gif-sur-Yvette, France), using a NextSeq 500/550 High Output v2 kit (Illumina). Raw data were processed using bcl2fastq2-2.18.12 (demultiplex), Cutadapt 3.2 (adapter trimming), FastQC v0.11.5 (quality control), resulting in 58-90 M reads (72 M reads in average per sample post-adapter trimming). In total, 87.5% of the sequences could be aligned with STAR (v2.7.10b; options: –sjdbGTFtagExonParentTranscript Parent) on the *Pimephales promelas* genome/transcriptome (GCA_016745375.1 with NCBI annotation release 100 for the genes definition). 76.4% of these alignments were assigned to genes using featureCounts (subreadds v1.5.2; options: -p -C -t gene). All raw sequences have been deposited in the Sequence Read Archive repository under accession number PRJNA1076136.

#### Identification of human and zebrafish orthologs

All putative proteins corresponding to retrieved fathead minnow genes were subjected to tBlastn analysis against the NCBI peptide sequences of zebrafish (Ensembl version 104, genome reference GRCz11) and human (Ensembl version 104, genome reference GRCh38p13) to generate for each fathead minnow gene a corresponding zebrafish best Blast hit and human genome nomenclature committee identifiers (HGNC IDs) (a.k.a. official gene symbols). For genes with several isoforms, the one encoding for the longest protein was chosen and used as bait for Blast analysis.

#### Identification of differentially expressed genes

Pre-processing checks and identification of any potential outliers was performed through graphical analysis, including hierarchical clustering and PCA plots. Differentially expressed genes between IFN-treated WT EPC-EC cells or *viperin*^*−/−*^ EPC-EC-Vip-C7 cells and their respective non-treated controls (ie. IFN vs Ctrl for each cell line) and between *viperin*^*−/−*^ EPC-EC-Vip-C7 compared to WT EPC-EC cells at the steady state or following IFN treatment (ie. KO vs WT for each treatment condition), were identified. Differentially expressed genes were identified using DESeq2 R package [50]. *p*-values were adjusted for multiple testing using Benjamini–Hochberg procedure. Genes were considered differentially expressed if they met the following criteria: adjusted *p* value < 0.05; log2fold change > 1 (upregulated genes) or < -1 (downregulated genes).

#### Gene set enrichment analysis

For functional gene set enrichment, Gene Ontology (GO) analysis and Kyoto Encyclopedia of Genes and Genomes (KEGG) pathway analysis were performed on DEGs using the web interface DAVID [51]. The predicted GO terms and KEGG pathways were based on the lists of official gene symbols corresponding to fathead minnow DEGs without using expression or fold change values. In order to identify effects on the pathways, up- and downregulated DEGs were inputted into DAVID separately. The same lists of official gene symbols were also analyzed with Ingenuity Pathway Analysis (IPA, Qiagen).

### rVHSV-Tomato fluorescence monitoring

The replication of rVHSV-Tomato in infected cell lines was monitored by sequential fluorescence measurement. WT EPC-EC, *viperin*^*−/−*^ EPC-EC-Vip-C7 and EPC-EC-Vip-C11 cells were seeded in 96-well plates to a final density of 1 × 10^5^ cells/well in L-15 medium supplemented with 2% heat-inactivated FBS and penicillin (100 U/mL)-streptomycin (100 μg/mL) and incubated overnight at 20 °C. The next day, the medium was removed and the cells were infected in octuplicates with 100 µL of rVHSV-Tomato diluted in L-15 without Phenol Red (Gibco) supplemented with 2% heat-inactivated FBS and penicillin (100 U/mL)-streptomycin (100 μg/mL) to reach MOI 0.1, 1 or 10 or left uninfected. At 24, 32, 48, 56, 72, 80 and 96 h post-infection, the tomato red fluorescence was measured using a fluorometer (Tecan Infinite M200PRO) with excitation and emission wavelengths of 548 and 593 nm, respectively. The fluorescence values were corrected by subtracting the mean values obtained from the non-infected wells.

### Statistical analysis

Apart from RNA-seq analysis, results shown in each figure were derived from at least two independent experiments; the data presented are means ± standard deviation (SD). Statistical tests used are indicated in the legend of each figure. All statistical analyses were performed using GraphPad Prism software version 9.5.1. For RNA-seq data, the statistical tests used in this study are included in the “RNA-seq analysis” section.

## Results

### Comparative analysis indicates that the genome of fathead minnow likely contains a unique *viperin* gene

**Fig. 1:**
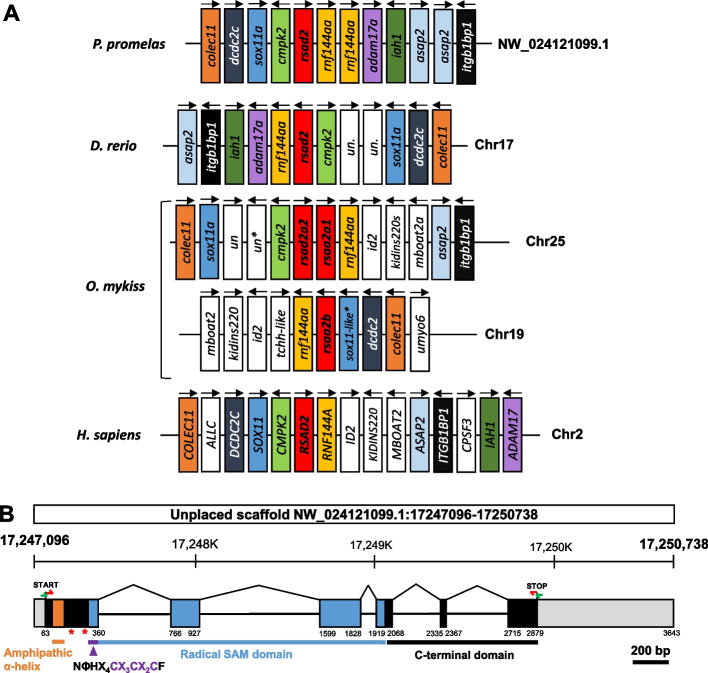
Synteny and genomic location of the likely unique *viperin* gene in the fathead minnow genome. **A** Synteny analysis of *viperin* loci in fathead minnow (*Pimephales promelas*, LOC120476724, unplaced scaffold NW_024121099.1, EPA_FHM_2.0), zebrafish (*Danio rerio*, LOC570456, GRCz11), rainbow trout (*Oncorhynchus mykiss*, LOC100135876, LOC110504183, LOC110498119, USDA_OmykA_1.1) and human (*Homo sapiens*, LOC91543, GRCh38.p13). The synteny was predicted using information extracted from recently released NCBI reference genomes. **B** Exon/intron structure of *P. promelas viperin* gene. Boxes represent exons and straight lines represent introns; grey boxes denote untranslated regions while colored and black boxes denote translated regions. Exonic parts encoding the N-terminal amphipathic α-helix domain (orange), the central radical SAM domain (blue) and the invariant motif responsible for binding Fe-S cluster (purple), which is included in a longer motif conserved among all ddhNTP synthases are represented. The location of each predicted domain or motif was obtained using SMART (Simple Modular Architecture Research Tool). The location of sgRNA-Vip1 and sgRNA-Vip2 is indicated by a red star. Nested PCR primers, used to amplify the *viperin* CDS are indicated by green and red arrows

Although a single *viperin* gene has been found in mammals and birds, reported numbers of *viperin* paralogs can vary from one to three in bony fish. For instance, tBlastn analysis revealed the presence of a unique *viperin* gene in fugu (*Takifugu rubripes*, LOC101074024), Japanese medaka (*Oryzias latipes*, LOC101175536), and zebrafish (*Danio rerio*, LOC570456). In contrast, in salmonid species, whose common ancestor has undergone a whole genome duplication event [52], two *viperin* paralogs are located on distinct loci in species belonging to the genus *Salmo*, including Atlantic salmon (*S. salar*, LOC100195910, LOC106566099) and brown trout (*S. trutta*, LOC115162541, LOC115172835) and three paralogs in species belonging the genus *Oncorhynchus*, including rainbow trout (*O. mykiss*, LOC100135876, LOC110504183, LOC110498119) (Fig. 1A), chinook salmon (*O. tshawytscha*, LOC112256495, LOC112255730, LOC112262031), sockeye salmon (*O. nerka*, LOC115138320, LOC115138321, LOC115146395) and coho salmon (*O. kisutch*, LOC109903880, LOC109903881, LOC109894649). Two of the three paralogs are tandemly arranged in head-to-tail orientation on the same chromosome, suggesting that they resulted from an independent tandem duplication event specific to the genus *Oncorhynchus*.

A unique *viperin* sequence (LOC120476724) is present in the current genome assembly EPA_FHM_2.0 (NCBI RefSeq assembly GCF_016745375.1) of the fathead minnow (*Pimephales promelas*), located on the unplaced scaffold NW_024121099.1. A 874-pb EST sequence (GH713605.1) covering 76% of the CDS from the predicted transcript (XM_039667881.1) with 100% identity supports the assembly and shows that the gene is expressed. The existence of a single *viperin* gene in fathead minnow is further supported by the presence of a unique ortholog in closely related cyprinid species, including zebrafish (*Danio rerio*; LOC570456), amur ide (*Leuciscus waleckii*; FLSR01004878:5,743,602–5746539), and grass carp (*Ctenopharyngodon idella*; LOC127498383); importantly these genes are located in a well-conserved synteny group (Fig. 1A). The sequence of the predicted protein XP_039523815.1 contains 345 amino acids and shares 70.9%, 70.8% and 68.6% identity with human VIPERIN (*Homo sapiens*, NP_542388.2), chicken Viperin (*Gallus gallus*, NP_001305372.2) and frog Viperin (*Xenopus tropicalis*, XP_002935073.2), respectively. These results confirm that this protein is highly conserved in teleosts as well as among vertebrates [7, 12].

To determine whether the *viperin* gene was present in the EPC-EC genome and expressed by these cells, nested PCR primers specific to the 5' and 3' untranslated regions (UTR) and to the 5’ and 3’-ends of the CDS (Fig. 1B) were used with cDNA from IPNV-infected EPC-EC cells and resulted in the amplification of a fragment of 1038 pb. This product matched the coding sequence of the *viperin* transcript (XM_039667881.1) predicted from the NCBI model with 100% identity (Fig. 1B). Structural domain analysis of the Viperin 345-aa polypeptide using SMART (Simple Modular Architecture Research Tool) [53] revealed the presence of a N-terminal transmembrane helix region (Phe13-Ile35) and a central radical SAM domain (Tyr61-Leu247), which comprises the canonical motif CX_3_CX_2_C [54–61] characteristic of the radical SAM superfamily (Fig. 1B). More specifically, the conserved motif NΦHX_4_CX_3_CX_2_CF (Φ being W, Y or F), recently described for all ddhNTP synthases [15] is also present in the sequence of fathead minnow Viperin (residues 60–75). Of note, the C-terminal tryptophane residue (W345), which is required for Viperin antiviral activity by playing a role in substrate recognition and/or interaction with partner proteins or cofactors such as cytosolic Fe/S protein assembly factor CIAO1 [4, 62], is also conserved in fathead minnow Viperin. This organization of functional domains is shared with the Viperin of other vertebrates [7, 12].

### *viperin* is induced following type I IFN stimulation and during viral infections in EPC-EC cells

The expression profile of *viperin* transcripts in EPC-EC cells in response to recombinant type I IFN and to viral infection was determined by RT-qPCR. For this purpose, two different viruses were used: viral hemorrhagic septicemia virus (VHSV), an enveloped negative-sense single-stranded RNA virus belonging to the genus *novirhabdovirus*, and infectious pancreatic necrosis virus (IPNV), a naked, double-stranded RNA virus belonging to the genus *aquabirnavirus*. To track the progression of the viral infection, we used a recombinant VHSV encoding the fluorescent protein tdTomato (rVHSV-Tomato) [42]. In addition to *viperin*, the expression pattern of *mx1*, another conserved type I IFN stimulated gene, was also examined for comparative purposes.

**Fig. 2:**
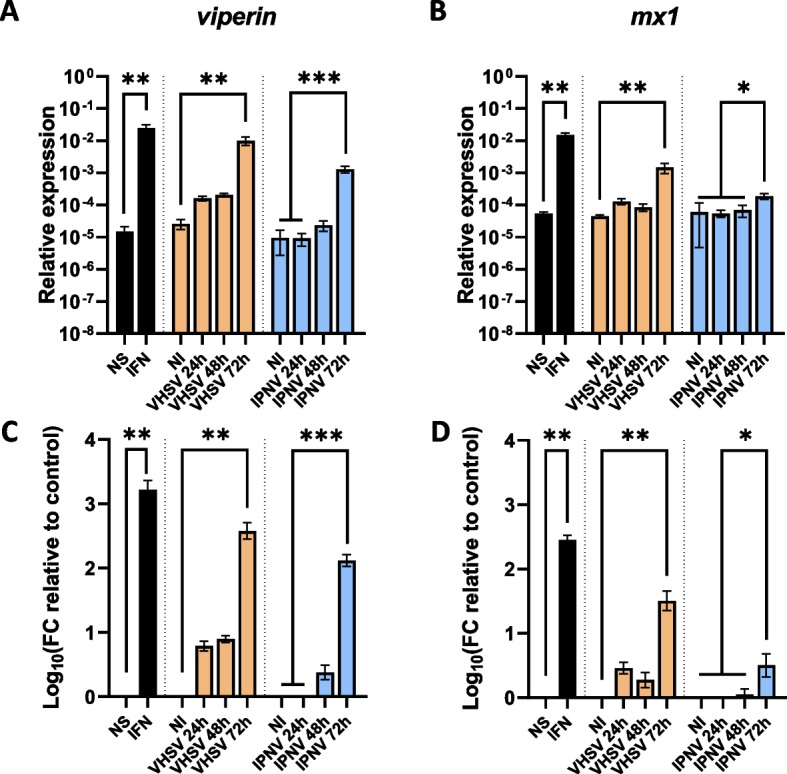
*viperin* and *mx1* expression in EPC-EC cells during type I IFN stimulation and viral infection. EPC-EC cells were stimulated with recombinant type I IFN (24 h), infected with rVHSV-Tomato (MOI 0.05) or IPNV31.75 (MOI 0.001) for 24 to 72 h post-infection, or left untreated (NI, non-infected; NS, non-stimulated). **A**,**B** Relative expression levels of *viperin* and *mx1* genes. **C**,**D** Fold change relative to non-stimulated or non-infected controls. Black bars show means ± SD from 2 pooled independent experiments (*n* = 3 for each experiment), orange bars show means ± SD (*n* = 4) and blue bars show means ± SD from 2 pooled independent experiments (*n* = 4 for each experiment), *, *p* < 0.05, **, *p* < 0.01, ***, *p* < 0.001, Kruskal–Wallis test with Dunn’s post-hoc multiple comparison tests

A strong induction of both *viperin* and *mx1* mRNA expression was observed at 24 h post-stimulation with recombinant fathead minnow type I IFNφ1 (3.2- and 2.5-logfold increase, respectively) compared to non-stimulated cells (Fig. 2). Similarly, both genes were significantly induced at 72 h post-infection with rVHSV-Tomato (2.6- and 1.5-logfold increase, respectively) (Fig. 2). In contrast, while *viperin* was also highly induced at 72 h post-infection with IPNV31.75 (3.1-logfold increase), *mx1* only displayed a weak but still significant induction at the same timepoint with this virus (0.5-logfold increase) (Fig. 2). Comparable results were previously obtained in the rainbow trout RTG-P1 cell line, where it was reported that IPNV suppressed the early activation of *mx* expression [63].

Altogether, our results show that *viperin* transcripts are strongly induced by both type I IFN and viral infections in EPC-EC cells. Furthermore, these observations support the use of EPC-EC as a parental cell line for the development of a *viperin*^*−/−*^ cell line to investigate the effects of its gene on responses to viral infection.

### CRISPR/Cas9-based edition of the *viperin* gene leads to null mutation and abolishes Viperin expression in EPC-EC cells

**Fig. 3:**
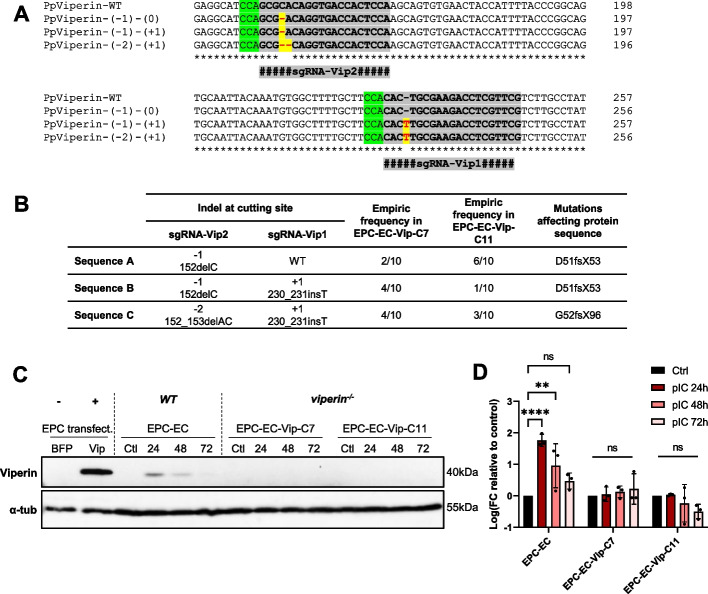
Development and validation of a *viperin*^*−/−*^ cell line. **A** Genotype of EPC-EC cells (WT) and EPC-EC-Viperin-C7 and -C11 clones obtained from sequencing of purified PCR products amplified from genomic DNA from each cell line and subcloned by TOPO TA cloning. The locations of the sgRNA-Vip1, sgRNA-Vip2 are highlighted in grey; the protospacer adjacent motif is in green and the indels in mutated sequences are in red highlighted in yellow. The corresponding amino acid sequences are available in Additional file 2. **B** Table summarizing the molecular characteristics of EPC-EC-Viperin-C7 and –C11 clones. **C** Validation of the *viperin* knockout by western blot. EPC-EC and EPC-EC-Viperin clones were stimulated with poly(I:C) (500 µg/mL) for 24-72 h; positive and negative controls are EPC cells transfected with pcDNA3.1-Hyg-BFP or pcDNA3.1-Hyg-BFP-P2A-Viperin, respectively. Cell lysates were separated by SDS-PAGE and immunoblotted with antibodies against Viperin and α-tubulin (α-tub). Full length blots are available in Additional file 3. **D** Densitometric quantification of (**B**). Viperin signal intensity normalized to α-tubulin signal intensity and graphed as fold change relative to non-stimulated cells. Bars show means ± SD from 3 pooled independent experiments; ns, non-significant, **, *p* < 0.01, ****, *p* < 0.0001, ordinary two-way ANOVA with Tukey’s post-hoc multiple comparison tests

To better understand the functions of fathead minnow Viperin, we disrupted the *viperin* gene in the EPC-EC cell line, using CRISPR/Cas9 technology. Two clonal cell lines deriving from FACS-sorted single cells, named EPC-EC-Vip-C7 and EPC-EC-Vip-C11, were further characterized. Both clones presented heterozygous mutations at the cutsites targeted by the two sgRNA used: a 1-nt deletion and 2-nt deletion at sgRNA-Vip2 target site and a partial 1-nt insertion at sgRNA-Vip1 target site, most likely affecting one haplotype only (Additional file 1). In order to fully genotype these clones, the PCR products comprising the sgRNA target sites were cloned into TOPO TA vectors and individual clones were sequenced. Surprisingly, the results revealed the presence of 3 distinct haplotypes from each clone (Fig. 3A,B), hereafter referred to as sequence A, sequence B and sequence C. Sequence A displayed a 1-nt deletion (152delC) at sgRNA-Vip2 cut site and no indel at sgRNA-Vip1 cut site (noted as -1/0); sequence B featured the same 1-nt deletion at sgRNA-Vip2 and a 1-nt insertion (230_231insT) at sgRNA-Vip1 cut site (noted as -1/ + 1) and sequence C had a 2-nt deletion (152_153delAC) at sgRNA-Vip2 cut site and a 1-nt insertion (230_231insT) at sgRNA-Vip1 cut site (noted as -2/ + 1). All indels resulted in frameshifts and in the appearance of premature stop codons at position 53 (D51fsX53 for sequences A and B) or at position 96 (G52fsX96 for sequence C) (Fig. 3B). Importantly, in sequence B, the frameshift at sgRNA-Vip2 generated a premature stop codon upstream the sgRNA-Vip1 ensuring an overall null mutation (Additional file 2). The existence of three and not just two different sequences can be explained in three ways: [1] each “clone” does not derive from a single cell; [2] there are at least two *viperin* paralog genes in the genome of fathead minnow; [3] the EPC-EC cell line and/or EPC-EC-Vip clones have undergone a local duplication event or (partial) chromosome gain during their respective development processes, resulting in more than two copies of the *viperin* gene.

As we could not exclude that these observations were due the presence of two highly similar *viperin* genes in the genome of EPC-EC cells, it was important to assess the expression of Viperin at the protein level. Preliminary western blot experiments showed that the expression of Viperin was induced in EPC-EC cells upon stimulation with poly(I:C), a synthetic dsRNA (data not shown). Therefore, we investigated the abolition of the Viperin expression in both EPC-EC-Vip-C7 and -C11 clones by western blot using poly(I:C) as an inducer. Our results showed that Viperin was induced in WT EPC-EC cells following exposure with poly(I:C) and its expression peaked as early as 24 h post-stimulation. In contrast, no Viperin signal was detected at any of the time points examined (24-72hpi) in *viperin*^*−/−*^ EPC-EC-Vip-C7 and -C11 clones (Fig. 3C,D, Additional file 3). Similar results were obtained using type I IFN supernatant as an inducer of Viperin expression (Additional file 4). These results confirmed that the expression of Viperin was effectively disrupted in both clones.

### *viperin* knockout has a significant impact on the cellular transcriptome regardless of its induction status

**Fig. 4:**
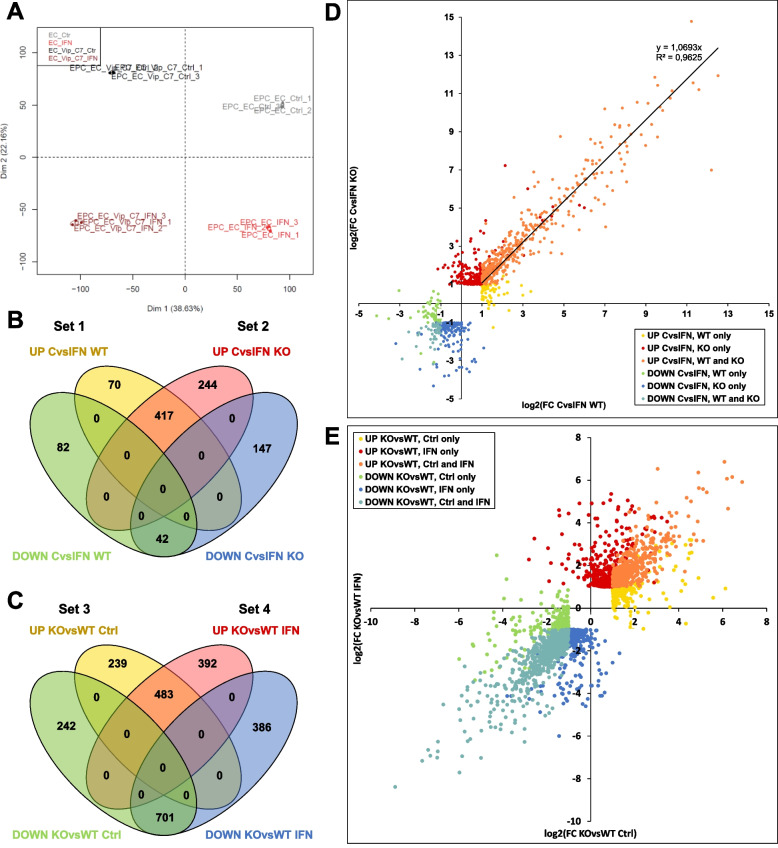
Comparison of DEGs in *viperin*^*−/−*^ and WT cell lines (steady state vs. IFN simulation). **A** Principal component analysis plot showing the distribution of all samples (*n* = 3 for each condition). Projection on the two first axes is shown (dimension 1: horizontal axis; dimension 2: vertical axis). **B** Venn diagram showing DEGs after IFN stimulation compared to non-stimulated condition (Ctrl) in the WT cell line (set 1) and in the *viperin*^*−/−*^ cell line (set 2). **C** Venn diagram showing DEGs in the *viperin*^*−/−*^ cell line compared to the WT cell line at the steady state (set 3) or following IFN simulation (set 4). Genes were considered DEGs if they met the following criteria: log2foldchange (FC) > 1 or < -1 and adjusted *p* value < 0.05. **D** Dotplot showing the fold change distribution of DEGs in the *viperin*^*−/−*^ cell line compared to the WT cell line at the steady state (x-axis) and following IFN treatment (y-axis). **E** Dotplot showing the fold change distribution of DEGs upon IFN treatment compared to non-stimulated condition in the WT cell line (x-axis) and in the *viperin*^*−/−*^ cell line (y-axis)

To explore the functions of Viperin, we used a whole transcriptome sequencing approach to compare gene expression in WT EPC-EC cells and *viperin*^*−/−*^ EPC-EC-Vip-C7 cells, at the steady state and following a 24 h treatment with type I IFN. To visualize the transcriptome response of WT EPC-EC and *viperin*^*−/−*^ EPC-EC-Vip-C7 to type I IFN, a principal component analysis (PCA) and hierarchical clustering were performed on gene expression datasets and revealed the clustering of individual samples into groups reflecting Viperin status (presence/absence) and stimulation status (non-stimulated/IFN-treated) (Fig. 4A, Additional file 5). In particular, the *viperin* knockout explains 38.6% of the variance (horizontal axis, dimension 1) while IFN treatment explains 22.2% of the variance (vertical axis, dimension 2). A total of 19,871 expressed genes were subjected to differential expression analysis, of which 18,955 were protein-coding genes (Additional file 6). The accuracy of the RNA sequencing and the resulting differential expression analysis were verified by assessing the expression of a few ISGs by RT-qPCR. The results showed the same expression pattern for all the genes examined, thereby validating the RNA-Seq data (Additional file 7).

In the following paragraphs, four different sets of DEGs were analyzed, depending on the type of comparison intended: [set 1] genes differentially expressed upon type I IFN treatment compared to non-stimulated condition (control) in the WT cell line; [set 2] genes differentially expressed upon type I IFN treatment compared to control condition in the *viperin*^*−/−*^ cell line; [set 3] genes differentially expressed in the *viperin*^*−/−*^ cell line compared to the WT cell line at the steady state (control condition); [set 4] genes differentially expressed in the *viperin*^*−/−*^ cell line compared to the WT cell line upon type I IFN treatment. In other words, sets 1 and 2 focus on the transcriptomic response to type I IFN in each cell line, respectively, while sets 3 and 4 highlight the impact of Viperin (presence/absence) on the cellular transcriptome at the steady state (*i.e.* without Viperin expression being induced) and following type I IFN stimulation (*i.e.* under Viperin induction condition).

Concerning sets 1 and 2, a large number of protein-coding genes were upregulated upon type I IFN treatment compared to the control condition in both cell lines (487 DEGs in the WT cell line and 661 DEGs in the *viperin*^*−/−*^ cell line) whilst fewer genes were downregulated (124 DEGs in the WT cell line and 189 DEGs in the *viperin*^*−/−*^ cell line) (Fig. 4B,D; Additional file 8). Of note, a large majority of genes upregulated upon IFN treatment (> 60%) are shared in both cell lines. In contrast, when comparing DEGs in the *viperin*^*−/−*^ cell line compared to the WT cell line (sets 3 and 4), more protein-coding genes were significantly differentially expressed both at the steady state and upon type I IFN treatment: 722 and 875 genes were more expressed in the *viperin*^*−/−*^ cell line compared to the WT cell line at the steady state and upon type I IFN treatment, respectively, while 943 and 1087 were less expressed in these same conditions, respectively (Fig. 4C,E; Additional file 8). Strikingly, more than 60% of the genes which are less expressed in *viperin*^*−/−*^ cells compared to WT are shared at the steady state and following IFN treatment and more than 55% of the genes which are more expressed in *viperin*^*−/−*^ cells than in WT are also common to both conditions, indicating that Viperin has a significant impact on the whole transcriptome, regardless of its induction status.

### Viperin does not modulate the canonical type I IFN response but likely plays a role in the modulation of the inflammatory response

To analyze the transcriptomic changes in both cell lines and identify the different pathways in which Viperin might be involved, we performed gene set enrichment for GO terms and KEGG pathways, based on official names (HGNC) of human orthologs of the differentially expressed fathead minnow genes, using the web interface DAVID [51].

#### Viperin does not modulate the canonical type I IFN response

Most GO terms were commonly enriched in the set of genes upregulated upon type I IFN treatment in the WT cell line (set 1) and in the *viperin*^*−/−*^ cell line (set 2) (Additional file 9). The 26 Biological Processes significantly enriched in the WT cell line were included in the 46 terms obtained from the *viperin*^*−/−*^ cell line. Many of these terms include generic GO terms associated with immune or inflammatory responses. In particular, the most significantly enriched terms (*ie*. with the lowest of *p* value) were “GO:0051607 ~ defense response to virus” and “GO:0045087 ~ innate immune response” in both cell lines. More specific terms related to IFN response ranked among the terms with the highest fold enrichment, including “GO:0002753 ~ cytoplasmic pattern recognition receptor signaling pathway” (26-fold enrichment in WT, 18-fold enrichment in *viperin*^*−/−*^ cell line), “GO:0060333 ~ interferon-gamma-mediated signaling pathway” (18-fold enrichment in WT, 14-fold enrichment in *viperin*^*−/−*^ cell line), “GO:0032727 ~ positive regulation of interferon-alpha production” (19-fold enrichment in WT, 13-fold enrichment in *viperin*^*−/−*^ cell line) and “GO:0032727 ~ positive regulation of interferon-alpha production” (13-fold enrichment in WT, tenfold enrichment in *viperin*^*−/−*^ cell line). Consistent results were obtained from the KEGG pathway analysis, where “hsa04623:Cytosolic DNA-sensing pathway”, “hsa04622:RIG-I-like receptor signaling pathway” as well as virus-specific pathways (e.g. “hsa05160:Hepatitis C”, “hsa05164:Influenza A”) were among the most enriched pathways (Additional file 10). These results confirm that the type I IFN treatment was effective and further suggest that the IFN response was similar in both cell lines.

**Fig. 5:**
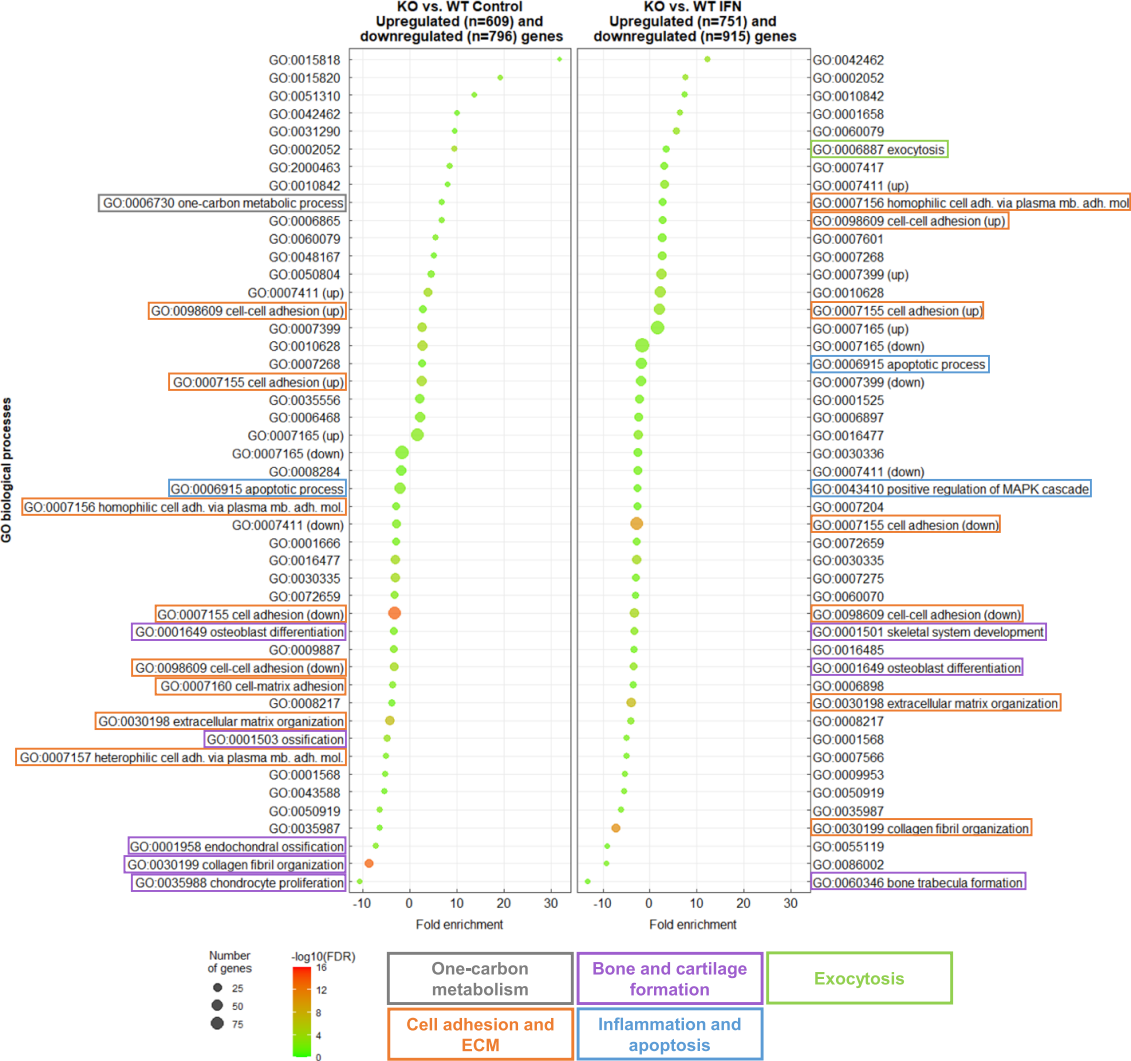
Gene enrichment suggests Viperin is involved in distinct biological processes in non-induced and induced conditions. Gene ontology analysis from the lists of genes differentially expressed the *viperin*^−/−^ cell line compared to the WT at the steady state (left panel) and upon type I IFN stimulation (right panel). GO terms have been filtered to show results with a Benjamini statistical score < 0.05. The size of the dot represents the number of genes involved within each biological process; colors indicate -log10 (False Discovery Rate) and colored boxes represent biological functions

However, DAVID analysis is only based on the lists of official gene symbols without using expression or fold change values. Therefore, it does not take into account a potential differential amplitude in gene expression between the two cell lines. In addition, differences of expression between multiple fish paralogs sharing a unique human ortholog cannot be analyzed in this way. To investigate whether the response magnitude to IFN treatment was different between the two cell lines, a linear regression was performed on the fold changes obtained for the 417 upregulated genes in both cell lines (Fig. 4D) revealing a regression coefficient of 1.0693 (R^2^ = 0.9625). This observation shows that the intensity of the *ISG* response is remarkably similar between/in the two cell lines, suggesting that Viperin does not have a significant global impact on the modulation of this response. Consistent with this observation, no GO terms associated with the innate immune response were enriched in the lists of genes differentially expressed in the *viperin*^*−/−*^ cell line compared to WT following type I IFN (Fig. 5, right panel). For further confirmation, we compared the lists of genes differentially expressed in the *viperin*^*−/−*^ cell line versus WT following type I IFN with a list of genes modulated by IFNφ1 in zebrafish larvae [64] (Additional file 11). Once again, very few genes were shared with the latter, further supporting that Viperin does not modulate the type I IFN response. It is therefore likely that *viperin* is essentially an effector gene in this pathway in EPC-EC cells.

#### Viperin acts as a regulator of the inflammatory response

**Fig. 6:**
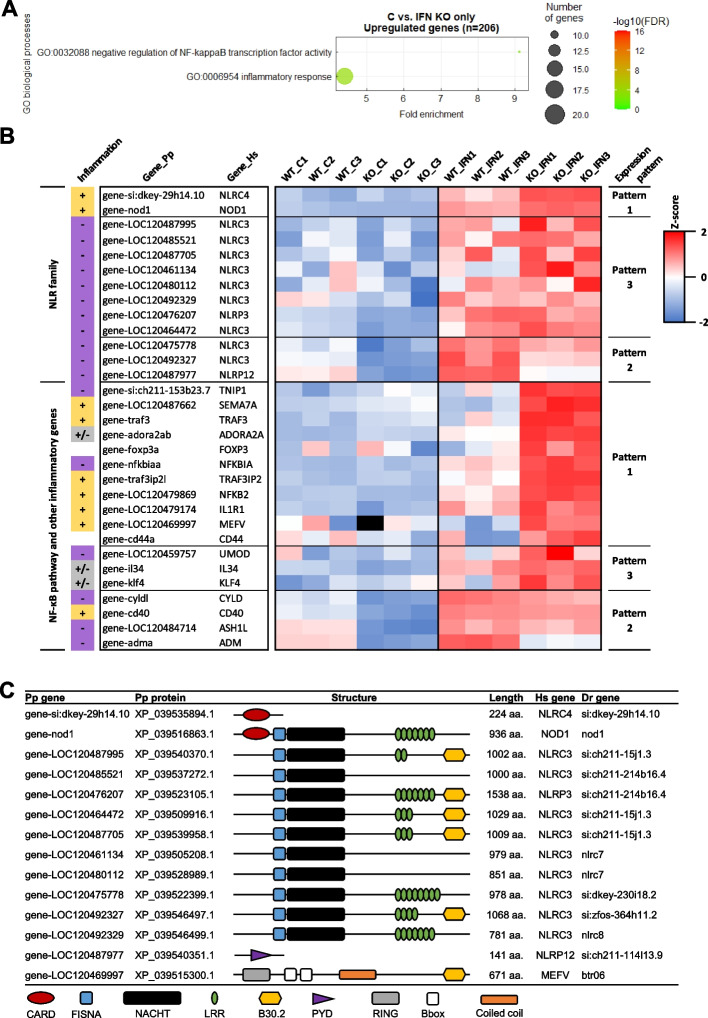
Viperin modulates the inflammatory response by downregulating pro-inflammatory genes and upregulating NF-κB pathway regulators. **A** Gene ontology analysis obtained from the list of genes exclusively upregulated in the *viperin*^−/−^ cell line following type I IFN treatment. GO terms have been filtered to show results with a Benjamini statistical score < 0.05. The size of the dot represents the number of genes involved within each biological process; colors indicate -log10 (False Discovery Rate) and colored boxes represent biological functions. **B** Heatmap of genes associated to the selected GO terms in **A**. Colors from blue to red represent the Z-score, which was calculated on a gene-by-gene basis by subtracting the overall mean of the log-transformed counts across all samples from the log-transformed count value of each gene, and then dividing that result by the overall standard deviation. Z-scores were calculated to ensure that the expression patterns were not overwhelmed by the expression values. Pattern 1 corresponds to genes showing no expression difference between both cell lines at the steady state but a higher induction in the *viperin*^*−/−*^ cell line compared to the WT upon IFN treatment; pattern 2 corresponds to genes that are less expressed in the *viperin*^*−/−*^ cell line versus WT at the steady state and upon type I IFN treatment but show higher fold change (Ctrl vs. IFN) in the *viperin*^*−/−*^ cell line; pattern 3 corresponds to the genes that show no significant expression difference between both cell lines at the steady state and upon type I IFN stimulation but still display a higher fold change (Ctrl vs. IFN) in the *viperin*^*−/−*^ cell line. Purple and yellow boxes indicate the anti- and pro-inflammatory functions known for the mammalian genes. Full-length heatmaps are available in Additional file 12. (**C**) Schematic representation of the structural domains of the NLRs listed in (**B**). CARD = Caspase recruitment domain, FISNA = Fish-specific NACHT associated domain, LRR = leucine-rich repeat (LRR), PYD = Pyrin domain, RING = RING-type zinc finger domain, Bbox = B-Box-type zinc finger domain, B30.2 = PRY-SPRY domain

Although both cell lines share a majority of DEGs following IFN treatment, a fair share of genes are exclusively modulated in one of them. In particular, 70 and 244 genes were exclusively upregulated in the WT and the *viperin*^*−/−*^ cell line, respectively, while 81 and 147 genes were downregulated in the WT or the *viperin*^*−/−*^ cell line only (Fig. 4B,D). GO analysis of these sublists did not result in significantly enriched pathways except for the list of genes that are exclusively upregulated in the *viperin*^*−/−*^ cell line upon IFN stimulation. Furthermore, for the other sublists, visual curation and IPA analysis did not lead to the identification of genes or pathways of interest. For the genes exclusively upregulated in the *viperin*^*−/−*^ cell line upon type I IFN, two GO terms were significantly enriched: “GO:0032088 ~ negative regulation of NF-kappaB transcription factor activity” (9.1-fold enrichment) and “GO:0006954 ~ inflammatory response” (4.4-fold enrichment) (Fig. 6A). To further analyze the role of Viperin in the inflammatory response, the specific genes identified as being enriched in this pathway were extracted and their expression levels were represented in a heatmap ((Fig. 6B). Interestingly, this subset of genes include members of the NOD-like receptors (NLR) family, involved in the formation of signaling platforms (including inflammasomes and nodosomes) of the inflammatory response [65]; genes involved in both canonical and non-canonical NF-κB pathways; as well as other genes playing a role in the inflammatory response.

These genes can be classified into three categories depending on their expression pattern in the *viperin*^*−/−*^ compared to the WT cell line: “pattern 1” includes genes (e.g. *NOD1*, *NFKBIA*, *NFKB2*, *IL1R1*) displaying no expression difference at the steady state but a higher induction in the *viperin*^*−/−*^ cells compared to WT upon IFN treatment; “pattern 2” corresponds to genes (e.g. *CYLD*, *CD40*, *ADM*) that are significantly less expressed in *viperin*^*−/−*^ cells versus WT at the steady state and upon IFN treatment, but that display a higher fold change (Ctrl vs. IFN) in the *viperin*^*−/−*^ cells; “pattern 3” corresponds to the genes (e.g. most *NLRC3* genes, *UMOD*, *IL34*) that show no significant expression difference between both cell lines at the steady state and upon type I IFN stimulation but still display a significant upregulation (Ctrl vs. IFN) in the *viperin*^*−/−*^ cells. Pattern 1 highlights genes that are downregulated by Viperin upon IFN treatment only, pattern 2 reveals genes that are upregulated by Viperin at the steady state while their induction is mitigated by Viperin upon type I IFN treatment; pattern 3 shows genes of which expression can be modulated by Viperin, but not in all conditions. This suggests that Viperin modulates the expression of inflammatory genes in distinct and complex ways.

NLRs proteins encoded by genes whose expression is modulated by Viperin have diverse structural domain compositions, which have been described in fish [66] (Fig. 6C). Indeed, mammalian NLRs are typically composed of an N-terminal effector domain, a central nucleotide-binding domain (NACHT) and a C-terminal ligand-binding region that comprises several leucine-rich repeats (LRRs) [65]. Members of the NLR family have been classified based on their N-terminal effector domain: for instance, NOD1-2 exhibit a N-terminal CARD domain; NLRP1-14 present a pyrin domain, NLRC3-5 feature an untypical CARD or unknown effector domain [65]. Although only a few of these NLRs-encoding genes, including NOD1-2 and NLRC3, present a direct ortholog in fish genomes, fish NLRs have also expanded into very large families of hundreds of proteins [66]. Several of them are characterized by the presence of a C-terminal B30.2 domain, which is typically present in some tripartite motif containing (TRIM) and Pyrin proteins [66]. Interestingly, in EPC-EC cells, Viperin-modulated NLRs present diverse domain combinations (CARD, LRR, B30.2), which excludes the modulation of a single type of NLRs by Viperin. The genes annotated as NLRC4 and NLRP12 only present a partial structure (CARD and PYD, respectively), hence are not classified within the canonical NLR family. Finally, the gene wrongly annotated *mefv* (aka. *pyrin*, another inflammasome) presents a typical TRIM structure (Fig. 6C).

It is noteworthy that most of the *NLRs* genes modulated by Viperin are homologous to mammalian NLRC3, which is a non-inflammasome-forming NLR member that negatively regulates inflammation by inhibiting NF-κB activation [67, 68]. NLRC3-like genes mainly follow expression patterns 2 and 3, suggesting that Viperin promotes their expression at the steady state but may limit their induction upon type I IFN. In contrast, *NOD1*, which promotes the inflammatory response by triggering the NF-κB and/or the MAPK pathways [69], follows expression pattern 1, indicating that Viperin downregulates its expression upon IFN treatment only.

In addition to NLRs, Viperin downregulates the expression of proinflammatory genes, including *IL1R1*, encoding the IL1β receptor. Of note, the fathead minnow gene annotated as *IL1R1* is homologous to the zebrafish CABZ01054965.1 gene, which was reported to be a functional ortholog for human *IL1RL2* [70]. IL1RL2 was shown to mediate IL-36-driven activation of NF-κB and to promote the secretion of proinflammatory chemokines and cytokines in epithelial tissues, likely in a similar fashion as IL-1α/β and IL-1R1 [71]. Furthermore, genes involved in both canonical and non-canonical NF-κB pathways (*TRAF3*, *TRAF3IP2*, *NFKB2*), which promote inflammation, were also more expressed in the *viperin*^*−/−*^ cell line compared to WT upon type I IFN treatment (pattern 1). Other genes involved in the inflammatory response and following expression pattern 1 include genes with dual inflammatory functions, such as *ADORA2a* [72], as well as a few anti-inflammatory genes, such as *TNIP1* (aka. TNFAIP3 interacting protein 1), which is an inflammation repressor that regulates NF-κB signaling [73] and *NFKBIA*, which inhibits the activity of dimeric NF-κB/Rel complex [74]. In addition, *CYLD*, which encodes a deubiquitinase that down-regulates NF-κB activation and limits inflammation [54], and *ADM*, which encodes an anti-inflammatory peptide [55], both follow expression pattern 2, suggesting that Viperin promotes their expression at the steady state but limit their induction upon type I IFN.

Taken together, these results suggest that Viperin downregulates the expression of pro-inflammatory genes, including *NOD1*, *IL1R1* as well as intermediate molecules and regulators of the NF-κB pathways, upon type I IFN treatment. Viperin also seems to modulate the expression of negative regulators of NF-κB activation (including *NLRC3*, *CYLD*) depending of its induction status: it may promote their expression at the steady state but limit their induction upon type I IFN.

#### rVHSV-Tomato does not replicate better in *viperin*^*−/−*^ cell lines

**Fig. 7:**
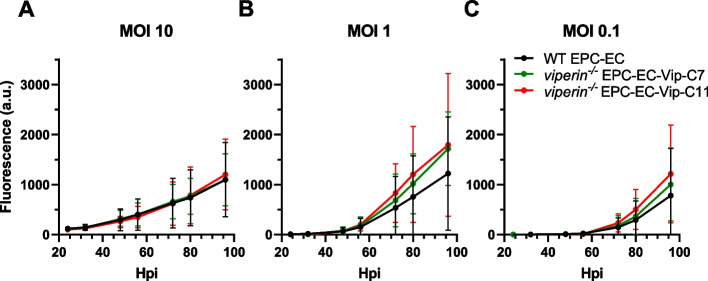
The *viperin* knockout has no significant impact on rVHSV-Tomato replication. EPC-EC (WT), EPC-EC-Vip-C7 and EPC-EC-Vip-C11 (*viperin*^*−/−*^) cells were infected with rVHSV-Tomato at **A** MOI 10, **B** MOI 1 or **C** MOI 0.1 and fluorescence was measured at different time points post-infection. Graphs show means ± SD from 6 independent experiments (*n* = 8 for each experiment)

Although Viperin does not seem to have a global impact on the IFN response in EPC-EC cells, we still assessed its antiviral role upon VHSV infection, as rVHSV-Tomato infection leads to a strong induction of *viperin* in this cell line (Fig. 2). To investigate the effect of Viperin on virus replication, we used rVHSV-Tomato, in which an expression cassette encoding tdTomato was inserted in the N-P intergenic region of VHSV genome [42]. As a consequence, tdTomato protein is only expressed during the replication cycle of the virus and fluorescence measurement can be used as a non-invasive indicator of viral replication. The evolution of the red fluorescence was sequentially monitored in WT EPC-EC and *viperin*^*−/−*^ EPC-EC-Vip-C7 and -C11 cell lines from 24 to 96 h post-infection. Remarkably, the fluorescence signal was not significantly different in both *viperin*^*−/−*^ clones compared to the WT cell line at any of the time points and MOI examined, suggesting that the knockout of *viperin* does not favor the replication of VHSV in this cell line (Fig. 7).

In addition, complementary experiments performed at lower MOIs (starting from MOI = 0.05) revealed that there was no difference in the appearance of CPE between WT EPC-EC cells and *viperin*^*−/−*^ EPC-EC-Vip-C7 and -C11 clones infected with tenfold serial dilutions of VHSV (data not shown). These results further indicate that the *viperin* knockout does not have a drastic effect on VHSV replication.

### Gene set enrichment analysis shows that Viperin may be involved in multiple pathways in addition to the type I IFN response

To further explore the functional role of Viperin, we analyzed the results of gene set enrichment on DEGs in the *viperin*^*−/−*^ cell line compared to the WT cell line, by combining analyses of set 3 (steady state) and set 4 (upon IFN treatment) (Fig. 5). These two datasets highlight DEGs specifically modulated by the presence/absence of Viperin, under physiological conditions *i.e.* when *viperin* transcripts are weakly expressed (steady state, set 3); and under pathological conditions *i.e.* when *viperin* transcripts are highly expressed (IFN treatment, set 4), respectively. In other words, analysis of both sets helps identify a potential regulatory role of Viperin in either treatment condition (*i.e.* role dependent on the induction status of Viperin), or in both (*i.e.* constitutive role, regardless of induction status).

#### Viperin modulates ECM organization and cell adhesion

Several GO terms were commonly enriched in both data sets regardless of the treatment. For downregulated DEGs, these shared GO terms fall into the large category of cellular adhesion and extracellular matrix (ECM) (Fig. 5), such as “GO:0030199 ~ collagen fibril organization” (8.7-fold enrichment in *viperin*^*−/−*^ cell line compared to the WT cell line at the steady state; 7.2-fold enrichment upon IFN treatment) and “GO:0030198 ~ extracellular matrix organization” (4.1-fold enrichment at the steady state; 3.9-fold enrichment upon IFN treatment). A few GO terms associated to this category were also specifically found in either treatment condition, such as “GO:0007160 ~ cell–matrix adhesion” (3.6-fold enrichment) and “GO:0007157 ~ heterophilic cell–cell adhesion via plasma membrane cell adhesion molecules” (5.0-fold enrichment), which were specifically enriched in downregulated DEGs at the steady state.

**Fig. 8:**
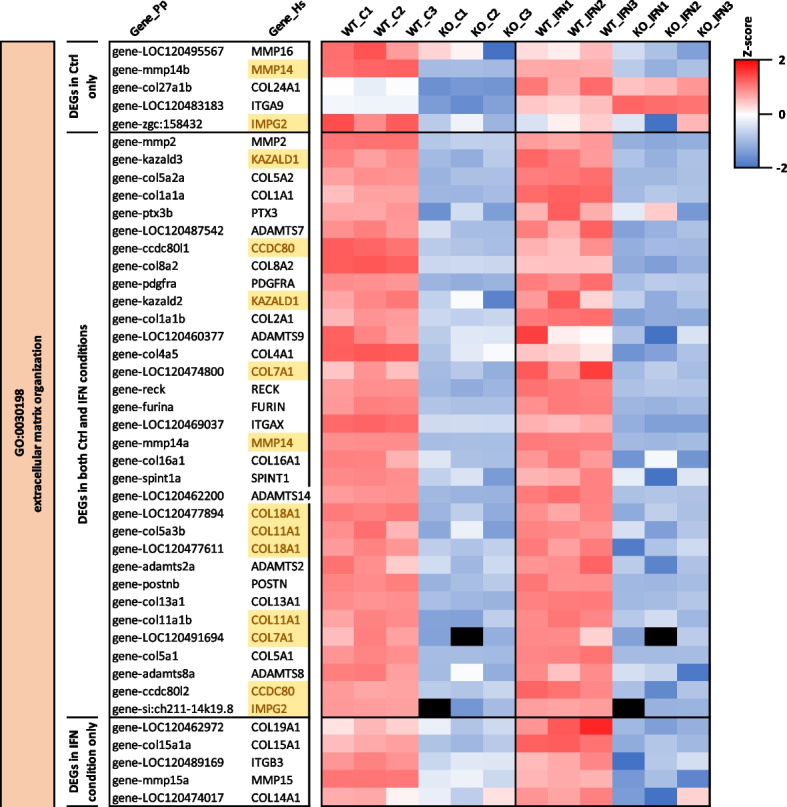
Viperin modulates the extracellular matrix organization regardless of its induction status Heatmap of genes associated to GO:0030198 extracellular matrix organization and differentially expressed in the *viperin*.^*−/−*^ cell line compared to the WT cell line at the steady state and upon IFN treatment. Colors from blue to red represent the Z-score (defined in Fig. 5); human genes highlighted in yellow indicate duplicates. Full-length heatmaps are available in Additional file 12

The ECM is a non-cellular network of macromolecules that are essential for many fundamental cellular functions, including structural support, cell adhesion, cell-to-cell communication and differentiation [56]. It is mainly composed of proteoglycans, fibrous proteins (including collagens and fibronectin, which are primarily produced by fibroblasts and laminins, which are mainly specific to epithelial, endothelial, and mesenchymal cells) and other secreted globular proteins such as growth factors, cytokines and ECM-specific enzymes (metalloproteases, matrix crosslinking enzymes and their respective regulators) [57]. In our study, several genes encoding fibrillar collagen α-chains (e.g. *COL1A1, COL2A1, COL4A1, COL5A1, COL5A2, COL11A1, COL13A1, COL16A1, COL18A1*) and non-collagenous proteins, such as laminins (*LAMA1, LAMA2, LAMA4*) and fibronectins (*FN1*) were found in the aforementioned enriched ECM-related GO terms (Fig. 8, Additional file 12). Other genes enriching either pathway include metalloproteases (including *ADAMTS7, ADAMTS2, ADAMTS14, MMP2, MMP14, TLL1, BMP1*), involved in the remodeling of the ECM [58], members of the lysyl oxidase (LOX) family (e.g. *LOX, LOXL2, LOXL3, LOXL4*) as well as regulators of matrix proteases (e.g. *RECK, SPINT1*), that are important for the assembly, structural organization, maintenance and homeostasis of the ECM [59].

As mentioned above, adhesion is one of the major biological functions of the ECM. ECM-cell adhesion is mediated by ECM transmembrane receptors, such as integrins, which bind to several ECM components, such as laminins, collagens, and fibronectin via their extracellular domain, thereby forming hemidesmosomes or focal adhesions [60]. Furthermore, cell adhesion also involves cell–cell junctions, which are mainly mediated by the cadherins for adherens junctions and desmosomes, or by claudins and occludins for tight junctions [61]. In our study, the GO term “GO:0007155 ~ cell adhesion” obtained from the list of downregulated genes in the *viperin*^*−/−*^ cell line compared to the WT cell line in both treatment conditions ranked among the most significantly enriched terms (ie. with the lowest of *p* value) (Fig. 5). Besides collagen-encoding genes, modulated genes include most of the adhesion proteins mentioned above, including integrins (*ITGs*) (*e.g. ITGA2, ITGA6, ITGA8, ITGA9, ITGA10, ITGA11, ITGB3*), proteins belonging to the cadherin superfamily, such as cadherin 2 (*CDH2*) and protocadherins (e.g. *PCDH1, PCDHA2, PCDHAC2, PCDH10, PCDH17, PCDH18),* cadherin related proteins (*CDHR1, CDHR5*) as well as genes from the claudin (CLDN) family (*CLDN6, CLDN11, CLDN19*) (Additional file 12). Furthermore, genes coding adapter proteins, such as talins (*TLN2*), α-actinins (*ACTN2*), and catenins (*CTNND2*) which make the connection between the intracellular domains of ITG and CDH, respectively, and the cytoskeleton [75, 76] are also included in the lists of DEGs between WT and *viperin*^*−/−*^ cell lines. Finally, thrombospondins (*THBS1-4*), which are glycoproteins that play an essential role in regulating cell–cell and cell–matrix interactions [77], are also downregulated in the *viperin*^*−/−*^ cell line compared to the WT. Of note, KEGG pathway analysis revealed similar pathways enriched in the downregulated gene sets, including “hsa04512:ECM-receptor interaction” and “hsa04510:Focal adhesion” (Additional file 13). Visualization of DEGs imposed on top of the ECM-receptor interaction pathway (Additional file 14) illustrates the extent to which this pathway is modulated in the *viperin*^*−/−*^ cell line compared to the WT.

Altogether, these results suggest that Viperin promotes ECM organization and cell adhesion, mainly independently from its induction status.

#### Viperin is a positive regulator of bone and cartilage metabolism

Several GO terms related to bone and cartilage formation are also strikingly enriched in the lists of genes downregulated in the *viperin*^*−/−*^ cell line compared to the WT cell line, including “GO:0035988 ~ chondrocyte proliferation” (10.7-fold enrichment) and “GO:0001503 ~ ossification” (4.7-fold enrichment) in the control condition, “GO:0060346 ~ bone trabecula formation” (13.2-fold enrichment) and “GO:0001501 ~ skeletal system development” (3.3-fold enrichment) in the IFN stimulated condition and “GO:0001649 ~ osteoblast differentiation” shared in both conditions (3.3- and 3.5-fold enrichment, respectively) (Fig. 5). The ECM is known for playing a critical role in bone formation [78], a significant number of genes involved in ECM organization and cell adhesion are also found in bone-related GO terms, including collagens (in particular type I collagen encoded *COL1A1*) as well as genes encoding non-collagenous proteins, such as *MMPs* (e.g. *MMP2, MMP14*, *MMP16*) and *THBSs* (e.g. *THBS3*). Furthermore, several genes encoding bone morphogenetic proteins (BMPs), including *BMP3* and *BMP5*, were also among these downregulated genes. BMPs are secreted cytokines, members of the TGF-β superfamily, and integral components of the bone ECM involved in developmental processes and bone formation. They trigger activation cascades through receptor binding leading to the transcription modulation of target genes involved in developmental processes and bone formation [79]. Interestingly, several genes involved in the BMP signaling pathways were also downregulated in the *viperin*^*−/−*^ cell line compared to the WT cell line, including a few receptors of BMPs (*BMPR1B*), BMP antagonists such as Noggin (*NOG*) and Follistatins (*FST, FSTL1, FSTL4*), which inhibit BMP activity by direct binding to BMPs and/or to their respective cell surface receptors [80, 81], molecules involved in their signaling pathways such as mitogen activated protein kinases (MAPKs) (e.g. *MAPK11, MAP2K6*), as well as their downstream targets such are MSX transcription factors (*MSX2*) [79, 82]. Altogether, our results suggest that Viperin is involved in the modulation of a genes sets involved in bone metabolism, regardless of its induction status.

#### Viperin downregulates one-carbon metabolism

**Fig. 9:**
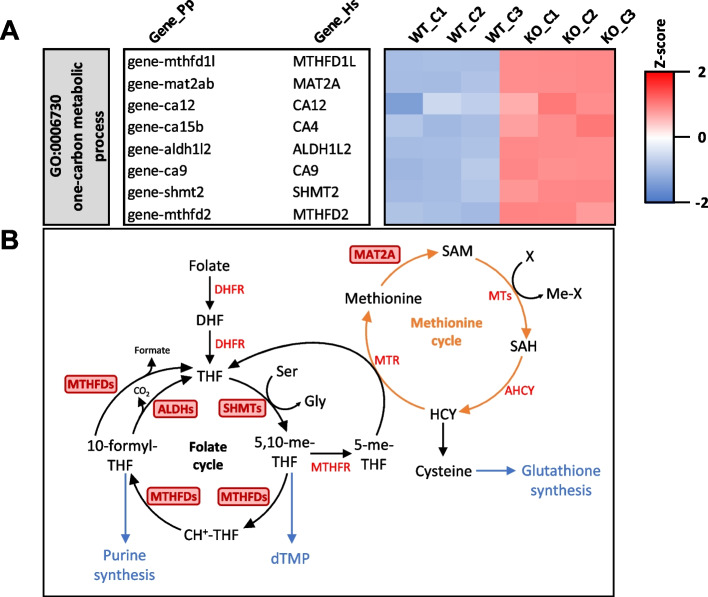
Viperin downregulates one-carbon metabolism. **A** Heatmap of genes associated to selected GO terms and differentially expressed in the *viperin*^*−/−*^ cell line compared to the WT cell line at the steady state. Colors from blue to red represent the Z-score (defined in Fig. 5). Full-length heatmaps are available in Additional file 12. **B** Schematic representation of one-carbon metabolism. 1C metabolism includes the methionine and folate cycles, which are central to multiple cellular functions. Metabolic intermediates are in black, enzymes are in red and red boxes indicate enzymes upregulated in the *viperin*^*−/−*^ cell line. DHF, dihydrofolate; THF, tetrahydrofolate; 5,10-me-THF (aka. 5,10-CH_2_-THF), 5,10-methylene-THF; CH^+^-THF, methenyl-THF; 10-formyl-THF (aka. 10-CHO-THF); SAM, S-adenosylmethionine; SAH, S-adenosylhomocysteine; HCY, homocysteine; dTMP, deoxythymidine monophosphate; DHFR, dihydrofolate reductase; SHMT, serine hydroxymethyl transferase; MTHFD, methylenetetrahydrofolate dehydrogenase; ALDH, aldehyde dehydrogenase; MTHFR, methylenetetrahydrofolate reductase; MTR, methionine synthase; MAT2A, methionine adenosyltransferase 2A; MT, methyl transferase; AHCY, adenosylhomocysteinase

The GO term “GO:0006730 ~ one-carbon metabolic process’ is enriched in the list of genes upregulated in the *viperin*^*−/−*^ cell line compared to the WT cell line in the control condition (Fig. 5). This term is of particular interest as one carbon metabolism results in the generation of SAM, which is a cofactor used by Viperin for the generation of ddhCTP. One carbon metabolism is a network of biochemical reactions that deliver one-carbon units (ie. methyl groups) to various biosynthetic pathways supporting biosynthesis of nucleotides (purines and thymidines), homeostasis of amino acids (glycine, serine, and methionine), epigenetic maintenance via histone methylation, and maintenance of redox balance (Fig. 9B) [83]. It comprises two interconnected metabolic pathways: the folate cycle and the methionine cycle. In the latter, methionine is converted into SAM by the methionine adenosyltransferase (MAT), encoded by *MAT2A/B*, in an ATP-dependent manner [84]. SAM is considered the main methyl donor in various biochemical reactions, including radical-mediated biochemical transformations; S-adenosylhomocysteine (SAH), the product of enzymatic extraction of the methyl group from SAM is converted to homocysteine, which can be “recycled” to methionine for the cycle to continue. This process requires vitamin B12 as a cofactor and uses a one-carbon unit that can be sourced from the folate cycle (methyl-THF) [84]. In the *viperin*^*−/−*^ cell line, upregulated genes comprise *MAT2A*, which is directly involved in the generation of SAM, as well as enzymes from the folate cycle, including aldehyde dehydrogenases (*ALDHs*), serine hydroxymethyl transferases (*SHMTs*) and methylenetetrahydrofolate dehydrogenases (*MTHFDs*) (Fig. 9). Of note, the KEGG pathway “hsa00270:Cysteine and methionine metabolism”, which is directly connected to one-carbon metabolism, is also enriched in the *viperin*^*−/−*^ cell line compared to the WT (4.2-fold enrichment), and specific genes involved in glutathione synthesis (e.g. cystathionine gamma-lyase (*CTH*), glutathione synthetase (*GSS*)) are upregulated (Additional file 13). These results suggest that in non-induced conditions but not after type I IFN stimulation, Viperin may act a negative regulator of the one-carbon metabolism, likely leading to reduced SAM generation via a negative feedback loop.

#### Viperin downregulates exocytosis

The GO term “GO:0006887 ~ exocytosis” is enriched in the list of genes upregulated in the *viperin*^*−/−*^ cell line compared to the WT cell line upon stimulation with type I IFN (Fig. 5). Exocytosis is a type of active bulk transport resulting in the fusion of a vesicle with the plasma membrane and the release of molecules into the extracellular space [85]. It involves vesicle trafficking along cytoskeleton filaments, vesicle tethering, vesicle docking and vesicle fusion with the plasma membrane. In the *viperin*^*−/−*^ cell line, upregulated genes are mainly involved in the regulation of vesicle exocytosis (e.g. *CADPS2, RIMS1, RIMS2*) or in vesicle tethering (e.g. *EXOC3L4*). These results indicate that Viperin might be involved in the regulation of exocytosis.

## Discussion

In this study, we have developed a fathead minnow epithelial-like cell line, in which the unique *viperin* gene has been knocked-out by CRISPR/Cas9 genome editing. Using a transcriptomic approach, we showed that in our model Viperin does not modulate the type I IFN response as many other ISG products do [86], suggesting that Viperin is only an effector gene of the type I IFN response *stricto *sensu. Our data indicate that it negatively regulates a number of genes involved in the inflammatory response, especially at steady state. In addition, Viperin appears to regulate the expression of key genes involved in multiple cellular processes, including one carbon metabolism, bone formation, extracellular matrix (ECM) organization and cell adhesion, even under non-pathological conditions.

### Are EPC-EC and/or EPC-EC-Vip cell lines aneuploid?

During the sequencing step in the development of the *viperin*^*−/−*^ clonal cell line, we identified three distinct genotyping sequences amplified by PCR from the gDNA of two isolated clones. The existence of three and not just two different sequences, corresponding in theory to each haplotype, can be explained in three ways: [1] each cell line does not derive from a single cell and is therefore not clonal and homogeneous; [2] there are at least two *viperin* paralog genes in the genome of fathead minnow; [3] the EPC-EC cell line and/or EPC-EC-Vip clones have undergone a local duplication event (tandem duplication), full chromosome gain (trisomy) or partial chromosome gain (e.g. partial trisomy following unbalanced translocation event, for instance) of the portion carrying the *viperin* gene during their respective development processes, resulting in more than two copies of the *viperin* gene. The first hypothesis is unlikely insofar as the clones were FACS-sorted and the sequencing of manually obtained subclones resulted in similar results (Additional file 15). Although the second hypothesis cannot be completely ruled out, in silico analysis of the most recent genome assembly strongly suggests that the fathead minnow genome only comprises a unique *viperin* gene, like the closely related species. The third hypothesis provides a fitting explanation for the three different sequences obtained from both EPC-EC-Vip-C7 and EPC-EC-Vip-C11 clones. Indeed, aneuploidy is a phenomenon relatively common in mammalian cell lines [87, 88] and several lines of evidence support the hypothesis that EPC-EC cells and/or its derivatives have undergone (partial) chromosome gain or local duplication during their respective development processes. Firstly, the three sequences obtained from the sequencing of both *viperin*^*−/−*^ clones can reflect the three haplotypes arising from a (partial) trisomy or from a duplication event affecting one copy of the *viperin* gene. Secondly, it has been shown that chromosome gain is often associated with impaired proliferation [89]. The EPC-EC cell line and all its derived clones have a much slower growth rate than the parental cell line EPC (data not shown), which is a phenomenon not observed in CHSE-EC cell line, deriving from CHSE-214 [44]. Assuming that this aneuploidy hypothesis is true, the question arises as to whether both EPC-EC cells and EPC-EC-Vip-C7 and -C11 or only the *viperin*^*−/−*^ clones are aneuploid, as it may have consequences on the transcriptomic data. We speculate that the event resulting in aneuploidy occurred during the development of the EPC-EC cell line and equally affects EPC-EC cells and their derivatives. Indeed, EPC-EC-Vip clones grow as slowly as EPC-EC cells. Furthermore, both *viperin*^*−/−*^ EPC-EC-Vip-C7 and -C11 clones present a “triple” genotype; because independent identical aneuploidy-resulting events (or local duplication events) are unlikely, this suggests that this phenomenon predates the cloning step. Finally, it has been previously shown in MEFs containing a single extra copy of a chromosome that the expression of genes located on the additional chromosome was proportional to the gene copy number (~ 1.5-fold increase in trisomic cells for the duplicated genes) [89]. In our case, the current fathead minnow genome (GCF_016745375.1, EPA_FHM_2.0) does not include chromosomes or linkage groups, which makes the analysis of additional (partial) chromosome(s) difficult. However, we assume that if (partial) chromosome gain happened during the development of the EPC-EC-Vip clones, it would be reflected in the expression of the genes located on the NW_024121099.1 containing the *viperin* gene. The analysis of the 925 genes located on NW_024121099.1 revealed that there were no significant and consistent fold change differences between EPC-EC-Vip-C7 and EPC-EC cells for both treatment conditions (Additional file 16). These results further support the hypothesis that if an event resulting in aneuploidy has affected the cells, it has most likely occurred during development of the parental line, EPC-EC.

Altogether, we propose that that both the EPC-EC cell line and its derivatives (EPC-EC-Vip-C7 and -C11) have more than one copy of *viperin*, resulting in the genotyping sequences observed.

### Role of Viperin in the IFN response and in the antiviral response

We have shown that Viperin does not globally modulate the amplitude of the canonical type I IFN response, suggesting that it mainly acts as an effector gene in the canonical type I IFN response in our epithelial fish cell line model. These results are not in line with previous published work, which reported that fish Viperin modulates the expression of some genes involved in IFN and inflammatory response [9, 38, 39]. However, these studies were based on overexpression approaches, which lead to unnaturally high levels of the protein of interest that may distort the effects of the endogenous protein. Alternatively, the use of type I IFN as an inducer of Viperin might also explain this discrepancy: indeed, Wang et al. found that overexpression of the splicing variant (lacking exon 5) but not the full length isoform of fathead minnow Viperin could induce the expression of RIG-I, IRF3 IRF7, type I IFN, MxA and PKR in FHM cells and this variant was only expressed upon infection with SVCV and not upon poly(I:C) stimulation [39]. Whether this variant can be expressed in our fathead minnow EPC-EC cell line is currently not known but the putative corresponding protein was not detected by Western blot upon stimulation with type I IFN supernatant (Additional file 4). Several studies on mammalian models did not provide unified results either concerning the role of Viperin on the regulation of the IFN response [25, 90, 91]. It was initially described that mammalian Viperin could promote the activation of key signaling mediators involved in the TLR7 and TLR9 pathways in plasmacytoid dendritic cells, thereby facilitating the production of type I IFN, but it was not involved in the production of type I IFN upon transfection with intracellular nucleic acids in MEFs [25]. In contrast, Viperin was found to act as a negative regulator of IFN-β induction in bone-marrow derived macrophages (BMDMs) upon poly(I:C) or 5’ppp-dsRNA transfection or type I IFN treatment [90]. These discrepancies may arise from the differences in cell types, inducers and/or assays used [92]. As a matter of fact, a recent study has reported that Viperin differentially modulated the induction of *ISGs* in a cell type- and inducer-dependent manner [91]. Altogether, our results show that, in epithelial cells, fathead minnow Viperin does not seem to have a major regulatory role on the expression of *ISGs* upon treatment with type I IFNs. Nonetheless, this observation does not exclude a role of fathead minnow Viperin in regulating the canonical IFN response in other cell types (dendritic cells, macrophages) and/or with another inducer (dsRNA, virus infection).

We observed no differences in fluorescence between *viperin*^*−/−*^ and WT cell lines infected with rVHSV-Tomato, suggesting that the *viperin* knockout did not result in higher replication of VHSV. In contrast, a recent study in *viperin*^*−/−*^ zebrafish larvae infected with rVHSV-ΔNV-EGFP reported a higher GFP signal and a tenfold increase in VHSV titer in *viperin*^*−/−*^ larvae compared to WT larvae (1.6 × 10^7^ vs 2.8 × 10^6^ TCID_50_/mL) [13, 41]. Taken together, these observations are consistent with the cell-type dependent role of Viperin in the antiviral response, leading to more complex effects in a whole organism.

### Role of Viperin in the inflammatory response

Although Viperin does not seem to be involved in the regulation of the IFN response, the functional analysis of our transcriptomic data revealed that a specific subset of proinflammatory genes were exclusively induced in the *viperin*^*−/−*^ cell line upon IFN stimulation, suggesting that Viperin might be a negative regulator of the inflammatory response. More specifically, our data revealed that Viperin modulates the expression of inflammatory genes in a complex manner: Viperin seems to downregulate the expression of specific pro-inflammatory genes upon type I IFN treatment and may also promote the expression of negative regulators of NF-κB activation at the steady state while limiting their induction upon type I IFN treatment.

In the literature, some studies have shown that Viperin enhanced the proinflammatory response [93, 94], while others have reported that it either did not modulate [25] or decreased the expression of proinflammatory genes [95]. Similarly to what is known about the role of Viperin in the IFN response, these studies suggest its contribution to the proinflammatory response may also be cell type- and treatment-dependent. In addition, the mechanisms by which it may modulate the pro-inflammatory response remain largely unknown. It was recently shown that the catalytic activity of nucleoside kinase CMPK2 is essential for NLRP3 inflammasome activation [96]. This nucleoside kinase functionally cooperates with Viperin, as it phosphorylates CDP into CTP, which is Viperin’s substrate [21]. It was suggested that CMPK2 proinflammatory function was linked to its capacity to enhance mitochondrial DNA synthesis via a mechanism that involves CTP synthesis [92, 96]. Because CTP is the preferential substrate for Viperin and it was proposed that CMPK2 and Viperin modulate the inflammatory response by increasing CTP production or consumption, respectively [92]. This hypothesis is supported by the observation that Viperin-mediated conversion of CTP into ddhCTP leads to the depletion of the mitochondrial pool of CTP [22].

Our transcriptomic data also point to some potential mechanisms leading the downregulation of the NF-κB pathways and other pro-inflammatory genes upon type I IFN treatment. In addition, Viperin seems to promote the expression of negative regulators of the NF-κB pathways, including several NLRC3-like genes, at the steady state. Of note, although mammalian NLRC3 has been shown to inhibit NF-κB activation via interactions with TRAF6, IRAK1 and/or TRAF3 [67, 68], the functional role of NLRC3-like genes in fish is still unclear and their large expansion makes their characterization even more challenging [66, 97].

Overall, this study highlights a role for Viperin in the inflammatory response that would be interesting to characterize in more detail in a future study. In particular, investigating the role of Viperin during bacterial infections could be an area for future research.

### Role of Viperin in other pathways

#### Role of Viperin in one-carbon metabolism

The gene set enrichment analysis of *viperin*^*−/−*^ cell line compared to WT revealed that at the steady state, Viperin may downregulate one carbon metabolism. One carbon metabolism encompasses both folate and methionine cycles and participates in the generation of SAM, a cofactor required for the enzymatic activity of Viperin [20]. We propose that Viperin might act as a negative regulator of one carbon metabolism under non-induced conditions, as a way to self-regulate the generation of ddhCTP. Indeed, although ddhCTP has been identified as a natural chain terminator of RNA-dependent RNA polymerase, endogenous ddhNTPs are small molecules with undefined functions [98]. Recent studies have explored the role of ddhCTP in cellular metabolism: Hsu et al. have shown that ddhCTP generated by Viperin can lead to the activation of the integrated stress response and inhibition of protein translation by enhancing ribosome collisions upon overexpression and during infection with West Nile virus [99]; Ebrahimi et al. have also provided evidence (albeit controversial) that ddhCTP was capable of inhibiting the enzymatic activity of NAD^+^-dependent enzymes [35]. Although the underlying mechanisms are still not well understood, it therefore appears that ddhCTP is not harmless to the cells and may affect their metabolism. It is tempting to speculate that, at least in non-infectious conditions, Viperin downregulates the generation of its cofactor, in order to limit the generation of ddhCTP when not needed. Nonetheless, because SAM is involved in a variety of metabolic processes, including DNA methylation, amino acid metabolism and transulfuration [83], it may have major consequences on the cellular metabolism. First and foremost, confirmation of this hypothesis would require quantification of the cellular concentration of SAM in the *viperin*^*−/−*^ cells compared to the WT.

#### Role of Viperin in cell adhesion and ECM

Intriguingly, our study suggests that Viperin positively modulates the expression of genes involved in cellular adhesion and ECM, including genes coding for structural proteins (collagens, fibronectin, laminin), ECM-specific enzymes and adhesion proteins (*e.g*. integrins, cadherins) among others. Interestingly, similar results were obtained in a very recent RNA-Seq study performed on 12Z endometriotic epithelial cells [100]: genes upregulated following Viperin overexpression were enriched for GO terms “ECM organization”, “cell-substrate adhesion”, “cell–matrix adhesion” and “collagen fibril organization” and opposite results were obtained upon *viperin* knockdown [100]. The identification of those enriched terms was not further discussed in this paper but it supports our findings and further suggests that our observations are not caused by a possible clonal effect. Altogether, these results shed light on a previously undiscovered function of Viperin. However, how Viperin modulates the expression of these genes remains to be determined.

#### Role of Viperin in bone metabolism

In our study, downregulated genes in the *viperin*^*−/−*^ cell line compared to the WT were also unexpectedly enriched for GO terms related to bone and cartilage formation. Although Viperin is not commonly associated with bone metabolism in the literature, a few studies have reported that Viperin is expressed in bone tissues and/or in bone or cartilage cells [31, 101]. In particular, the rat ortholog of *viperin* was highly expressed in differentiating primary osteoblasts in vitro as well in osteoblast progenitors and mature osteoblasts in sections of rat tibiae and in mechanically loaded bones [31]. More recently, *viperin* was identified in one of the QTLs explaining the size variation of Meishan pigs, suggesting that it might play a role in bone and skeletal development [102]. Viperin was also found to be involved in osteoclast differentiation [101] and chondrogenic differentiation [32]. Taken together, these results suggest that Viperin might be active as a regulator of cellular differentiation during cartilage and bone formation. However, the mechanisms by which Viperin modulates these metabolic processes are not well understood. Steinbusch et al. have shown that Viperin promotes the secretion of CXCL10, which in turn inhibits TGF-β/SMAD2/3 activity involved in chondrogenic differentiation [32]. Our study may provide another potential line of action, as many DEGs included genes involved in ECM organization and cell adhesion. The ECM is known for playing a key role in bone formation [78]; therefore, we propose that Viperin is involved in bone metabolism via modulating the expression genes involved in ECM and cellular adhesion. Consistent with the fact that EPC-ECs are epithelial cells, genes specifically expressed by bone-specific cells were not identified in our transcriptomic datasets. As a consequence, a regulatory role of Viperin on the expression of this specific gene subset could not be explored in this study.

## Conclusions

In conclusion, our transcriptomic analysis revealed that Viperin does not modulate the type I IFN response but may downregulate specific subsets of pro-inflammatory genes while upregulating negative regulators of the NF-κB pathways. It also appeared to play a role in regulating metabolic processes, including one carbon metabolism, bone formation, ECM organization and cell adhesion.

### Supplementary Information

Below is the link to the electronic supplementary material.

Additional file 1. Alignment of chromatograms from *viperin*^*-/-*^ EPC-EC-Viperin clones with EPC-EC (WT) cell line. Chromatograms showing edited and wild-type (control) sequences in the region around the sequences targeted by sgRNA-Vip1 and sgRNA-Vip2 from EPC-EC-Viperin-C7 and EPC-EC-Viperin-C11 (*viperin*^*-*/-^) clones. The horizontal black line represents the guide sequence; the horizontal red dotted line corresponds to the PAM site; the vertical black dotted line represents the actual cut site. The red and purple boxes show the inserted or deleted nucleotides in each edited clone. Alignments were obtained using Synthego ICE Analysis tool (v3). Note that for the reverse sequence from EPC-EC-Viperin-C11, ICE results could not be used due to the fact that the cut site was too close from sequence start; the alignment was done manually instead.

Additional file 2. Amino acid sequences corresponding to the mutated viperin sequences amplified from genomic DNA from WT EPC-EC and *viperin*^*-/-*^ EPC-EC-Viperin-C7 and -C11 and subcloned by TOPO TA cloning. The first amino acids affected by a frameshift are in red, the frameshifts are in green and the premature end of the polypeptides are represented by a red star. The immunogen peptide recognized by the anti-viperin antibody (PA5-42231, Invitrogen) is outlined in black.

Additional file 3. Original full-length blots used in Figure 3 to validate the *viperin*^*-*/-^ cell lines. Regions corresponding to the cropped images are surrounded by a dotted line.

Additional file 4. Validation of the *viperin* knockout by western blot using type I IFN supernatant as an inducer. EPC-EC and EPC-EC-Viperin clones were stimulated with recombinant type I IFN supernatant (1:10) for 24h; positive and negative controls are EPC cells transfected with pcDNA3.1-Hyg-BFP or pcDNA3.1-Hyg-BFP-P2A-Viperin, respectively. EPC-EC cells stimulated with poly(I:C) (500 µg/mL, 24h) were also included for comparison purposes. Cell lysates were separated by SDS-PAGE and immunoblotted with antibodies against Viperin. The red arrow indicates the Viperin protein.

Additional file 5. Descriptive analysis of RNAseq results from WT EPC-EC and *viperin*^*-/-*^ EPC-EC-Vip-C7 (KO) stimulated with type I IFN or left untreated (Ctrl). Euclidian clustering showing the distribution of all samples (*n*=3 for each condition).

Additional file 6. Tables showing differentially expressed transcripts in WT EPC-EC and *viperin*^*-/-*^ cell lines at the steady state and following type I IFN stimulation.

Additional file 7. Validation of RNA-Seq data by RT-qPCR analysis on a selected number of ISGs. The expression levels of the following genes were analyzed by RT-qPCR and compared to RNA-Seq data: *beta-actin *(gene-actb2, LOC120489986 and gene-actb1, LOC120463340), *mx1* (gene-mx1, LOC120468849), *viperin* (gene-rsad2, LOC120476724), *pkr* (gene-eif2ak2, LOC120460990) and *stat2* (gene-stat2, LOC120491376). Orange and blue bars represent RNA-Seq data and RT-qPCR results, respectively.

Additional file 8. Volcano plots showing differentially expressed genes in WT EPC-EC and *viperin*^*-/-*^ EPC-EC-Vip-C7 (KO) stimulated with type I IFN or left untreated (Ctrl). (A,B) Volcano plots showing DEGs after IFN stimulation compared to non-stimulated condition (Ctrl) in the WT cell line (A) and in the *viperin*^*-/-*^ cell line (B). (C,D) Volcano plots showing DEGs (log2foldchange (FC) > 1 or <-1, adjusted *p*.value < 0.05), in the *viperin*^*-/-*^ cell line compared to the WT cell line at the steady state (C) or following IFN simulation (D). Red dots represent upregulated genes while blue dots represent downregulated genes.

Additional file 9. Gene ontology analysis of DEGs upon IFN treatment compared to non-stimulated condition in the WT cell line (A) and in the *viperin*^*-/-*^ cell line (B). GO terms have been filtered to show results with a Benjamini statistical score <0.05. The size of the dot represents the number of genes involved within each biological process and colors represent -log10 (False Discovery Rate).

Additional file 10. KEGG pathway analysis of the DEGs upon IFN treatment compared to the control in the WT and in the *viperin*^-/-^ cell lines. KEGG pathway terms have been filtered to show results with a Benjamini statistical score <0.05.

Additional file 11. Venn diagram showing DEGs in the *viperin*^*-/-*^ cell line compared to the WT cell line following type I IFN treatment and previously identified IFNϕ1 modulated genes in zebrafish larvae. The list of IFNφ1 modulated genes comes from Levraud et al., 2019 (56). For comparison purposes, the the zebrafish best Blast hit corresponding to each DEG in the list (considered as the zebrafish ortholog) was used, explaining why some genes are found in both UP and DOWN categories.

Additional file 12. Full-length heatmaps of genes associated to selected GO terms.

Additional file 13. KEGG pathway analysis of the DEGs in the *viperin*^-/-^ cell line compared to the WT cell line at the steady state. KEGG pathway terms have been filtered to show results with a Benjamini statistical score <0.05. Red arrows indicate pathways detailed in Additional file 14.

Additional file 14. Modulation of ECM-receptor interactions in the *viperin*^*-/-*^ cell line compared to the WT cell line at the steady state (upper panel) and upon IFN stimulation (lower panel). DEG datasets were mapped onto the pathway using Pathview. Green and red colors show down- and upregulation, respectively.

Additional file 15. Genotyping results from EPC-EC-Vip-C7 and EPC-EC-Vip-C11 subclones. Indels at sgRNA-Vip2 cut site were analyzed using forward sequences with Synthego ICE analysis tool v3. Indels at sgRNA-Vip1 cutsite were manually analyzed using reverse sequences, as ICE was unable to perform the analysis due to the too short reading window around this cutsite. ICE KO-score indicates the proportion of indels leading to a frameshift; R² indicates how well the proposed distribution fits the sequence of the edited sample.

Additional file 16. Comparison of expression pattern of genes (*n*=925) located on the scaffold NW_0241210099.1 in EPC-EC-Vip-C7 cells compared to EPC-EC cells at the steady state (Ctrl) and following IFN treatment (IFN). No increase in “gene expression” was detected. ns, non-significant, one sample t-test.

## Data Availability

The RNA-seq datasets generated and analyzed during the current study are available in the Sequence Read Archive repository, under accession number PRJNA1076136.
