# Supplementary material for: Unexpected regulatory functions of cyprinid Viperin on inflammation and metabolism
Source: BMC Genomics. 2024 Jun 29;25:650. doi: 10.1186/s12864-024-10566-x (PMC11218377; doi:10.1186/s12864-024-10566-x)
Supplement: Supplementary file 1 — Additional file 1. Alignment of chromatograms from viperin-/- EPC-EC-Viperin clones with EPC-EC (WT) cell line. Chromatograms showing edited and wild-type (control) sequences in the region around the sequences targeted by sgRNA-Vip1 and sgRNA-Vip2 from EPC-EC-Viperin-C7 and EPC-EC-Viperin-C11 (viperin-/-) clones. The horizontal black line represents the guide sequence; the horizontal red dotted line corresponds to the PAM site; the vertical black dotted line represents the actual cut site. The red and purple boxes show the inserted or deleted nucleotides in each edited clone. Alignments were obtained using Synthego ICE Analysis tool (v3). Note that for the reverse sequence from EPC-EC-Viperin-C11, ICE results could not be used due to the fact that the cut site was too close from sequence start; the alignment was done manually instead. [file 12864_2024_10566_MOESM1_ESM.pdf]

>PpViperin-LOC120476724-exon1

ATGTTGATGCCATTGTGTTTCAAGGACGTCCACAGCTTATTTTCAGCCCTGTTGAGATGGATTTTGATGATGGTATCAGGCACACTGGTGTCTCTTGGGATGATCA
GTCGTCCAAAGATTCGCACCAGAGAGCAGAAAGAGGGAT***CCAGCGCACAGGTGACCACTCCA***AGCAGTGTGAACTACCATTTTACCCGGCAGTGCAAT
#####sgRNA-Vip2#####
TACAAATGTGGCTTTTGCTT***CCACACTGCGAAGACCTCGTTCG***TTTTGCCTATTGAAGAGGCAAAGCGAGGGTTACGACTTCTGAAAGAAGCAG
#####sgRNA-Vip1#####

Clones	sgRNA	Primers	ICE deconvoluted sequences
**EPC-EC-Vip-C7**	**sgRNA-Vip2**	Vip-gen-F	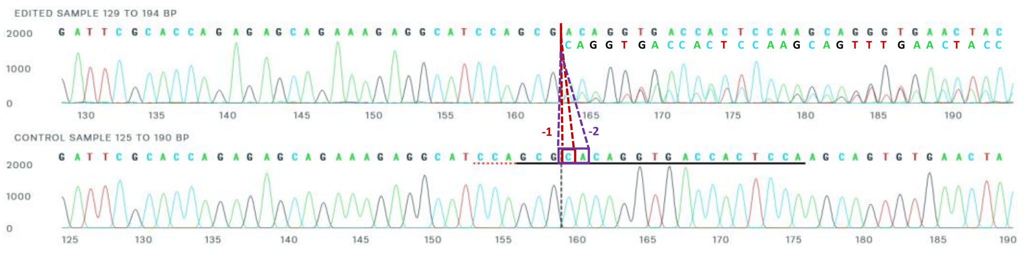
	**sgRNA-Vip1**	Vip-gen-R	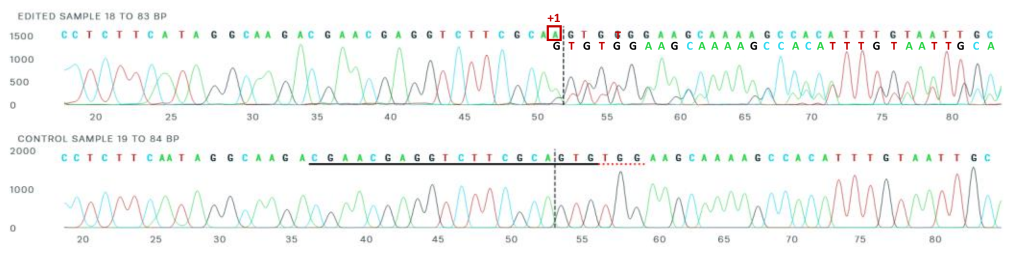
**EPC-EC-Vip-C11**	**sgRNA-Vip2**	Vip-gen-F	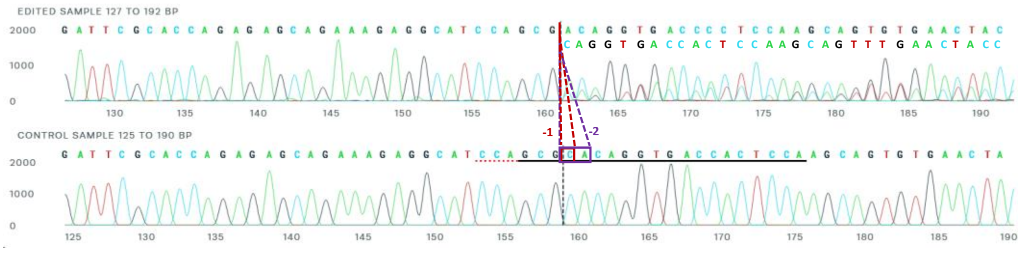
	**sgRNA-Vip1**	Vip-gen-R	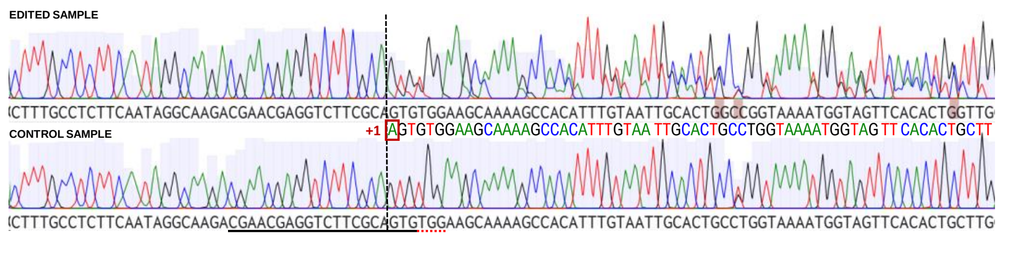

Additional file 1: Alignment of chromatograms from *viperin*$^{-/-}$ EPC-EC-Viperin clones with EPC-EC (WT) cell line.

Chromatograms showing edited and wild-type (control) sequences in the region around the sequences targeted by sgRNA-Vip1 and sgRNA-Vip2 from EPC-EC-Viperin-C7 and EPC-EC-Viperin-C11 (*viperin*$^{-/-}$) clones. The horizontal black line represents the guide sequence; the horizontal red dotted line corresponds to the PAM site; the vertical black dotted line represents the actual cut site. The red and purple boxes show the inserted or deleted nucleotides in each edited clone. Alignments were obtained using Synthego ICE Analysis tool (v3). Note that for the reverse sequence from EPC-EC-Viperin-C11, ICE results could not be used due to the fact that the cut site was too close from sequence start; the alignment was done manually instead.
