# Supplementary material for: Unexpected regulatory functions of cyprinid Viperin on inflammation and metabolism
Source: BMC Genomics. 2024 Jun 29;25:650. doi: 10.1186/s12864-024-10566-x (PMC11218377; doi:10.1186/s12864-024-10566-x)
Supplement: Supplementary file 2 — Additional file 2. Amino acid sequences corresponding to the mutated viperin sequences amplified from genomic DNA from WT EPC-EC and viperin-/- EPC-EC-Viperin-C7 and -C11 and subcloned by TOPO TA cloning. The first amino acids affected by a frameshift are in red, the frameshifts are in green and the premature end of the polypeptides are represented by a red star. The immunogen peptide recognized by the anti-viperin antibody (PA5-42231, Invitrogen) is outlined in black. [file 12864_2024_10566_MOESM2_ESM.pdf]

```
>PpViperin-WT
MLMPLCFKDVHSFFSALLRWILMMVSGTLVSLGMISRPKIRTREQKEASSAQVTTPSSVNYHFTRQCNYKCGFCFHTAKTSFVLPIEEAKRGLRLLKEAG
MEKINFSGGEPFLHERGSFLGELVRYCKQELLLPSVSIVSNGSLIKESWFQKYGDYLDILAVSCDSFNEDTNKVIGRGQGKKSHLDNLHKVCSWCRDYKV
AFKINSVINTYNVDEDMTEQITALNPVRWKVFQCLLIDGENAGENSLREAEKFVISEQQFQDFLDRHKSVKCLVPESNQKMRDSYLILDEYMRFLDCREG
RKDPSKSVLDVGVEEAIKFSGFDEKMFLIRGGKYVWSKEDMKLEW

>PpViperin-(-1)-(0)
MLMPLCFKDVHSFFSALLRWILMMVSGTLVSLGMISRPKIRTREQKEASSDR*PLQAV*TTILPGSAITNVAFASTLRRPRSSCLLKRQSEGYDF*KKQE
WKKSTFQVESPFFMREALFWESWSDTANRSCCFRASASLVMAV*SKNPGFRNTVTTWTFLQYLAIVLTKTPIKSLAEVRARRAI*TICIKFVPGAGTTRW
LSKSTP*STPTMWTKI*QSRSLL*TQCAGRSSSVC*LMVKTLGRTASARQKNLSLVSSNSKTSWTAIRASSVWFQSLIKR*ETLT*FLMNICASWIAERGG
KIRQSPFWMLVWKRPSSSVVLMRRCSS*EGGNMCGARKI*NWSG

>PpViperin-(-1)-(+1)
MLMPLCFKDVHSFFSALLRWILMMVSGTLVSLGMISRPKIRTREQKEASSDR*PLQAV*TTILPGSAITNVAFASTLAKTSFVLPIEEAKRGLRLLKEAG
MEKINFSGGEPFLHERGSFLGELVRYCKQELLLPSVSIVSNGSLIKESWFQKYGDYLDILAVSCDSFNEDTNKVIGRGQGKKSHLDNLHKVCSWCRDYKV
AFKINSVINTYNVDEDMTEQITALNPVRWKVFQCLLIDGENAGENSLREAEKFVISEQQFQDFLDRHKSVKCLVPESNQKMRDSYLILDEYMRFLDCREG
RKDPSKSVLDVGVEEAIKFSGFDEKMFLIRGGKYVWSKEDMKLEW

>PpViperin-(-2)-(+1)
MLMPLCFKDVHSFFSALLRWILMMVSGTLVSLGMISRPKIRTREQKEASSADDHSKQCELPFYPAVQLQMWLLLPHLRRPRSSCLLKRQSEGYDF*KKQE
WKKSTFQVESPFFMREALFWESWSDTANRSCCFRASASLVMAV*SKNPGFRNTVTTWTFLQYLAIVLTKTPIKSLAEVRARRAI*TICIKFVPGAGTTRW
LSKSTP*STPTMWTKI*QSRSLL*TQCAGRSSSVC*LMVKTLGRTASARQKNLSLVSSNSKTSWTAIRASSVWFQSLIKR*ETLT*FLMNICASWIAERGG
KIRQSPFWMLVWKRPSSSVVLMRRCSS*EGGNMCGARKI*NWSG
```

Additional file 2: Amino acid sequences corresponding to the mutated viperin sequences amplified from genomic DNA from WT EPC-EC and *viperin*$^{-/-}$ EPC-EC-Viperin-C7 and -C11 and subcloned by TOPO TA cloning.

The first amino acids affected by a frameshift are in red, the frameshifts are in green and the premature end of the polypeptides are represented by a red star. The immunogen peptide recognized by the anti-viperin antibody (PA5-42231, Invitrogen) is outlined in black.
