# Supplementary material for: Unexpected regulatory functions of cyprinid Viperin on inflammation and metabolism
Source: BMC Genomics. 2024 Jun 29;25:650. doi: 10.1186/s12864-024-10566-x (PMC11218377; doi:10.1186/s12864-024-10566-x)
Supplement: Supplementary file 3 — Additional file 3. Original full-length blots used in Figure 3 to validate the viperin-/- cell lines. Regions corresponding to the cropped images are surrounded by a dotted line. [file 12864_2024_10566_MOESM3_ESM.pdf]

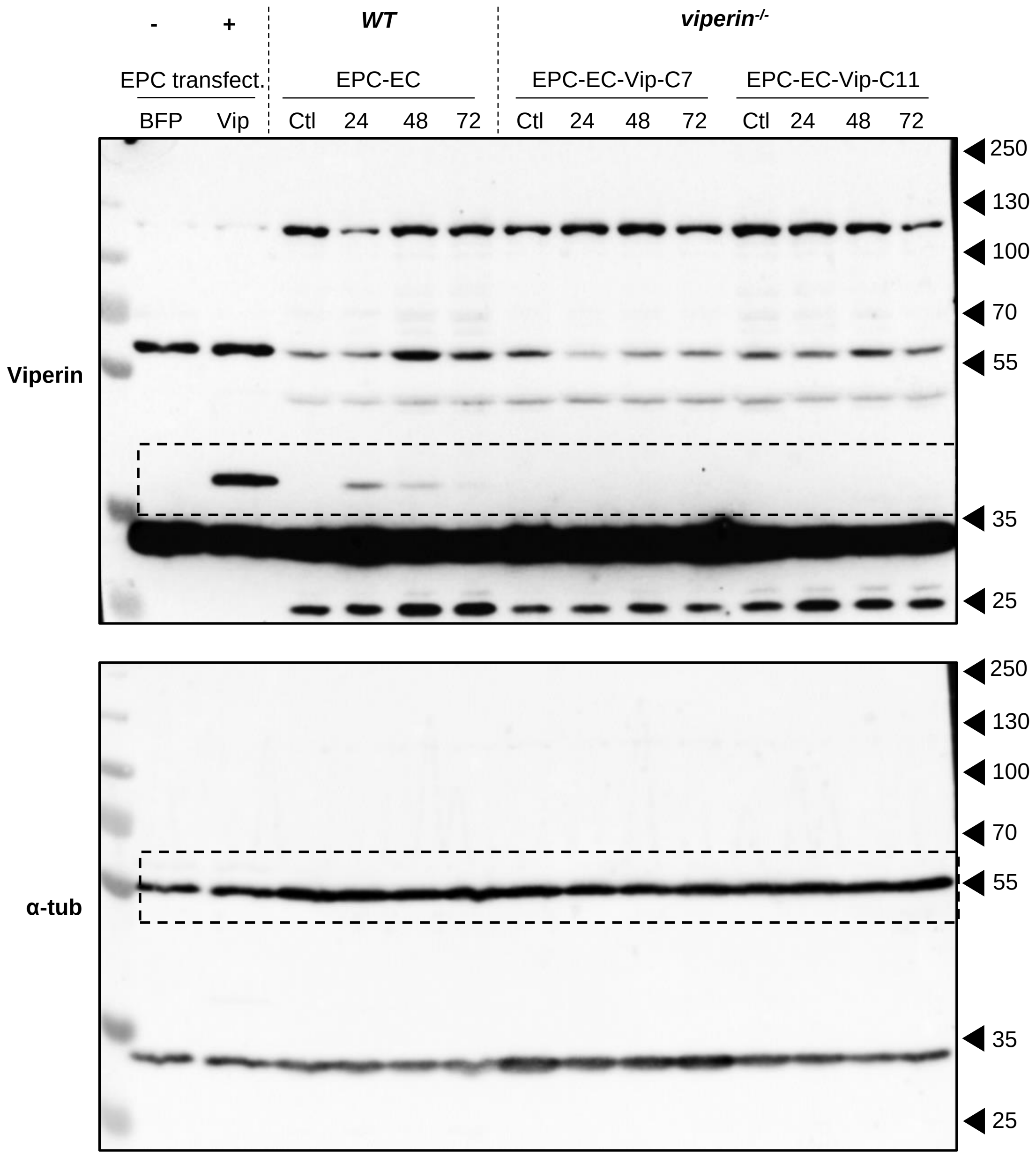

Additional file 3: Original full-length blots used in figure 1 to validate the *viperin*$^{-/-}$ cell lines.

Regions corresponding to the cropped images are surrounded by a dotted line.
