# Supplementary material for: Unexpected regulatory functions of cyprinid Viperin on inflammation and metabolism
Source: BMC Genomics. 2024 Jun 29;25:650. doi: 10.1186/s12864-024-10566-x (PMC11218377; doi:10.1186/s12864-024-10566-x)
Supplement: Supplementary file 4 — Additional file 4. Validation of the viperin knockout by western blot using type I IFN supernatant as an inducer. EPC-EC and EPC-EC-Viperin clones were stimulated with recombinant type I IFN supernatant (1:10) for 24h; positive and negative controls are EPC cells transfected with pcDNA3.1-Hyg-BFP or pcDNA3.1-Hyg-BFP-P2A-Viperin, respectively. EPC-EC cells stimulated with poly(I:C) (500 µg/mL, 24h) were also included for comparison purposes. Cell lysates were separated by SDS-PAGE and immunoblotted with antibodies against Viperin. The red arrow indicates the Viperin protein. [file 12864_2024_10566_MOESM4_ESM.pdf]

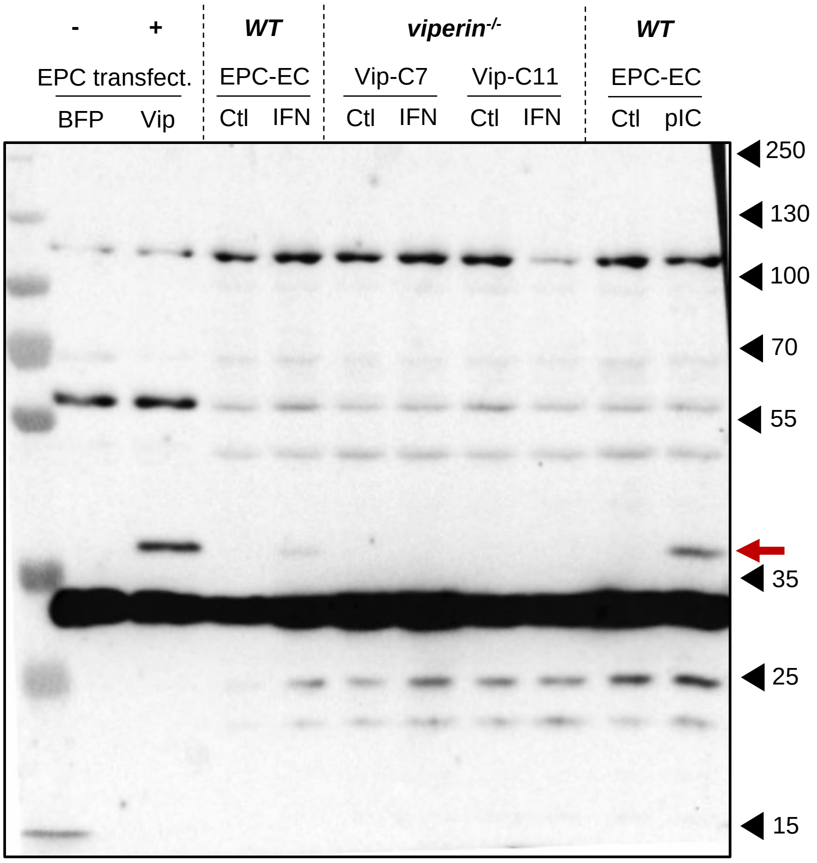

Additional file 4: Validation of the viperin knockout by western blot using type I IFN supernatant as an inducer.

EPC-EC and EPC-EC-Viperin clones were stimulated with recombinant type I IFN supernatant (1:10) for 24h; positive and negative controls are EPC cells transfected with pcDNA3.1-Hyg-BFP or pcDNA3.1-Hyg-BFP-P2A-Viperin, respectively. EPC-EC cells stimulated with poly(I:C) (500 µg/mL, 24h) were also included for comparison purposes. Cell lysates were separated by SDS-PAGE and immunoblotted with antibodies against Viperin. The red arrow indicates the Viperin protein.
