# Supplementary material for: Unexpected regulatory functions of cyprinid Viperin on inflammation and metabolism
Source: BMC Genomics. 2024 Jun 29;25:650. doi: 10.1186/s12864-024-10566-x (PMC11218377; doi:10.1186/s12864-024-10566-x)
Supplement: Supplementary file 5 — Additional file 5. Descriptive analysis of RNAseq results from WT EPC-EC and viperin-/- EPC-EC-Vip-C7 (KO) stimulated with type I IFN or left untreated (Ctrl). Euclidian clustering showing the distribution of all samples (n=3 for each condition). [file 12864_2024_10566_MOESM5_ESM.pdf]

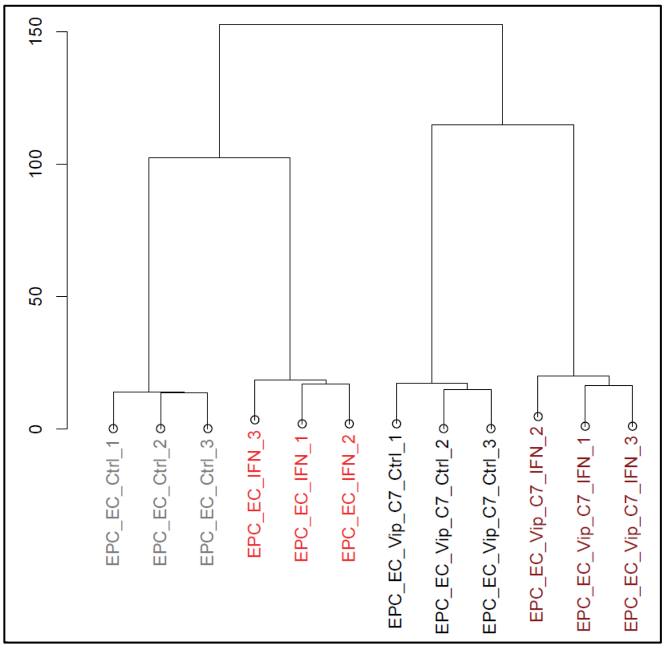

Additional file 5: Descriptive analysis of RNAseq results from WT EPC-EC and *viperin*$^{-/-}$ EPC-EC-Vip-C7 (KO) stimulated with type I IFN or left untreated (Ctrl).

Euclidian clustering showing the distribution of all samples (n=3 for each condition).
