# Supplementary material for: Unexpected regulatory functions of cyprinid Viperin on inflammation and metabolism
Source: BMC Genomics. 2024 Jun 29;25:650. doi: 10.1186/s12864-024-10566-x (PMC11218377; doi:10.1186/s12864-024-10566-x)
Supplement: Supplementary file 7 — Additional file 7. Validation of RNA-Seq data by RT-qPCR analysis on a selected number of ISGs. The expression levels of the following genes were analyzed by RT-qPCR and compared to RNA-Seq data: beta-actin (gene-actb2, LOC120489986 and gene-actb1, LOC120463340), mx1 (gene-mx1, LOC120468849), viperin (gene-rsad2, LOC120476724), pkr (gene-eif2ak2, LOC120460990) and stat2 (gene-stat2, LOC120491376). Orange and blue bars represent RNA-Seq data and RT-qPCR results, respectively. [file 12864_2024_10566_MOESM7_ESM.pdf]

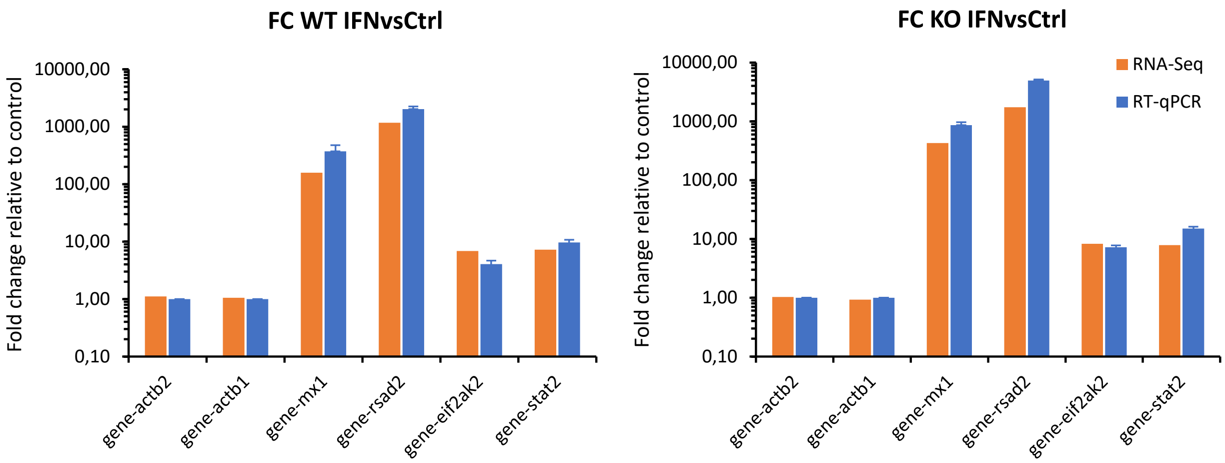

Additional file 7: Validation of RNA-Seq data by RT-qPCR analysis on a selected number of ISGs.

The expression levels of the following genes were analyzed by RT-qPCR and compared to RNA-Seq data: *beta-actin* (gene-actb2, LOC120489986 and gene-actb1, LOC120463340), *mx1* (gene-mx1, LOC120468849), *viperin* (gene-rsad2, LOC120476724), *pkr* (gene-eif2ak2, LOC120460990) and *stat2* (gene-stat2, LOC120491376). Orange and blue bars represent RNA-Seq data and RT-qPCR results, respectively.
