# Supplementary material for: Unexpected regulatory functions of cyprinid Viperin on inflammation and metabolism
Source: BMC Genomics. 2024 Jun 29;25:650. doi: 10.1186/s12864-024-10566-x (PMC11218377; doi:10.1186/s12864-024-10566-x)
Supplement: Supplementary file 8 — Additional file 8. Volcano plots showing differentially expressed genes in WT EPC-EC and viperin-/- EPC-EC-Vip-C7 (KO) stimulated with type I IFN or left untreated (Ctrl). (A,B) Volcano plots showing DEGs after IFN stimulation compared to non-stimulated condition (Ctrl) in the WT cell line (A) and in the viperin-/- cell line (B). (C,D) Volcano plots showing DEGs (log2foldchange (FC) > 1 or <-1, adjusted p.value < 0.05), in the viperin-/- cell line compared to the WT cell line at the steady state (C) or following IFN simulation (D). Red dots represent upregulated genes while blue dots represent downregulated genes. [file 12864_2024_10566_MOESM8_ESM.pdf]

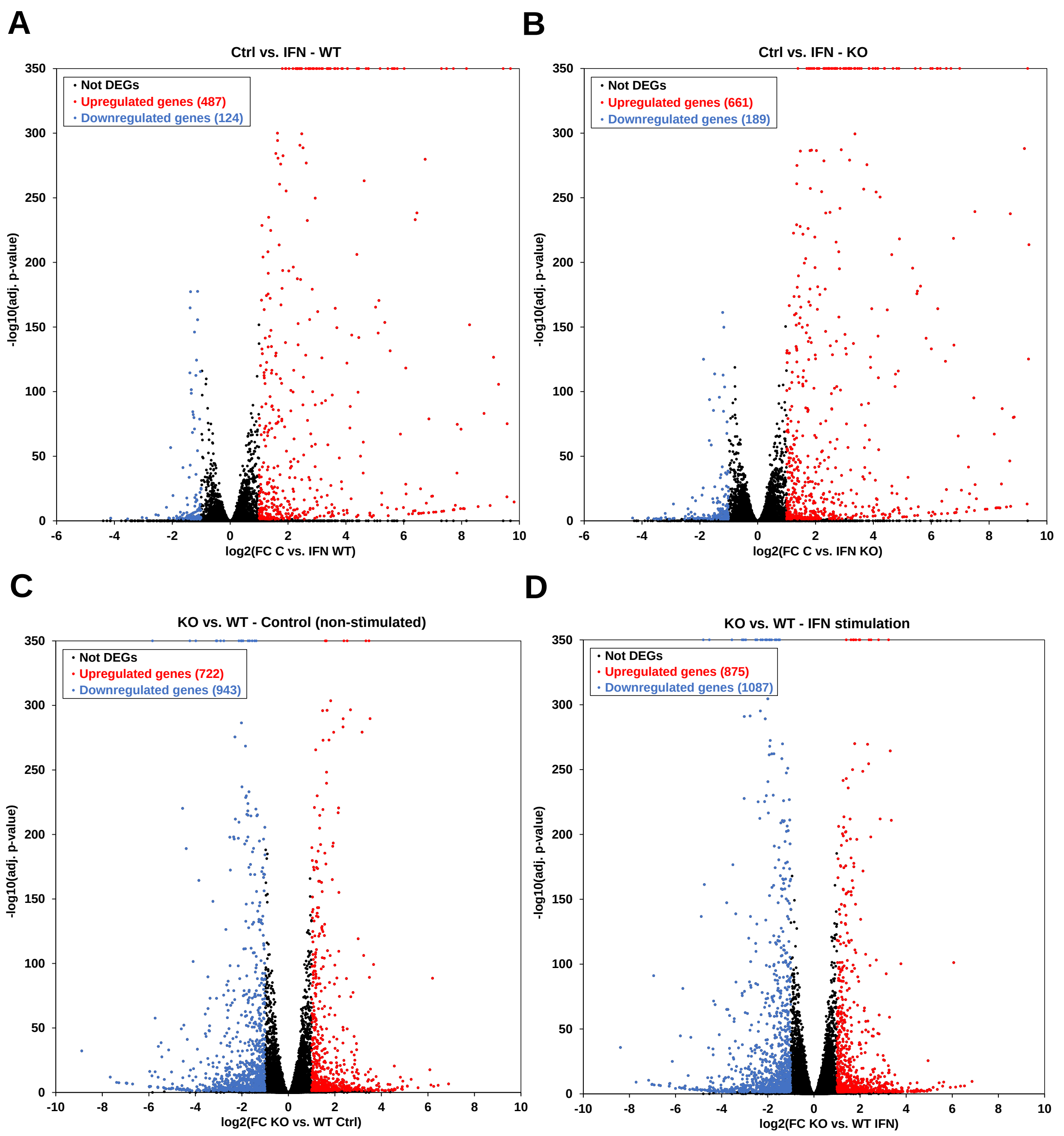

Additional file 8: Volcano plots showing differentially expressed genes in WT EPC-EC and *viperin*$^{-/-}$ EPC-EC-Vip-C7 (KO) stimulated with type I IFN or left untreated (Ctrl).

(**A,B**) Volcano plots showing DEGs after IFN stimulation compared to non-stimulated condition (Ctrl) in the WT cell line (A) and in the *viperin*$^{-/-}$ cell line (B). (**C,D**) Volcano plots showing DEGs (log2foldchange (FC) > 1 or <-1, adjusted p.value < 0.05), in the *viperin*$^{-/-}$ cell line compared to the WT cell line at the steady state (C) or following IFN simulation (D). Red dots represent upregulated genes while blue dots represent downregulated genes.
