# Supplementary material for: Unexpected regulatory functions of cyprinid Viperin on inflammation and metabolism
Source: BMC Genomics. 2024 Jun 29;25:650. doi: 10.1186/s12864-024-10566-x (PMC11218377; doi:10.1186/s12864-024-10566-x)
Supplement: Supplementary file 9 — Additional file 9. Gene ontology analysis of DEGs upon IFN treatment compared to non-stimulated condition in the WT cell line (A) and in the viperin-/- cell line (B). GO terms have been filtered to show results with a Benjamini statistical score <0.05. The size of the dot represents the number of genes involved within each biological process and colors represent -log10 (False Discovery Rate). [file 12864_2024_10566_MOESM9_ESM.pdf]

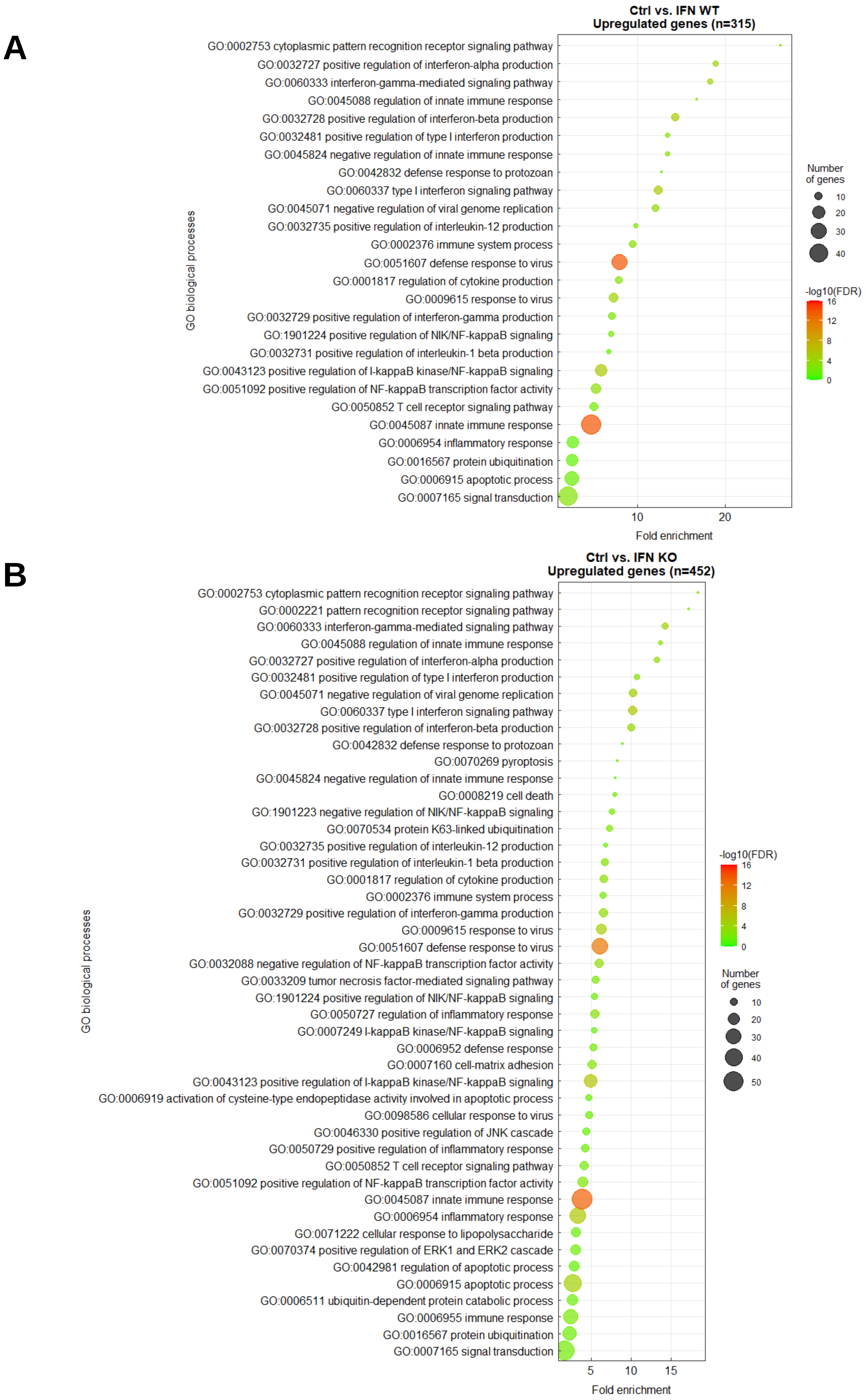
A
Ctrl vs. IFN WT
Upregulated genes (n=315)
GO:0002753 cytoplasmic pattern recognition receptor signaling pathway
GO:0032727 positive regulation of interferon-alpha production
GO:0060333 interferon-gamma-mediated signaling pathway
GO:0045088 regulation of innate immune response
GO:0032728 positive regulation of interferon-beta production
GO:0032481 positive regulation of type I interferon production
GO:0045824 negative regulation of innate immune response
GO:0042832 defense response to protozoan
GO:0060337 type I interferon signaling pathway
GO:0045071 negative regulation of viral genome replication
GO:0032735 positive regulation of interleukin-12 production
GO:0002376 immune system process
GO:0051607 defense response to virus
GO:0001817 regulation of cytokine production
GO:0009615 response to virus
GO:0032729 positive regulation of interferon-gamma production
GO:1901224 positive regulation of NIK/NF-kappaB signaling
GO:0032731 positive regulation of interleukin-1 beta production
GO:0043123 positive regulation of I-kappaB kinase/NF-kappaB signaling
GO:0051092 positive regulation of NF-kappaB transcription factor activity
GO:0050852 T cell receptor signaling pathway
GO:0045087 innate immune response
GO:0006954 inflammatory response
GO:0016567 protein ubiquitination
GO:0006915 apoptotic process
GO:0007165 signal transduction
GO biological processes
10
20
Fold enrichment
Number of genes
10
20
30
40
-log10(FDR)
16
12
8
4
0
B
Ctrl vs. IFN KO
Upregulated genes (n=452)
GO:0002753 cytoplasmic pattern recognition receptor signaling pathway
GO:0002221 pattern recognition receptor signaling pathway
GO:0060333 interferon-gamma-mediated signaling pathway
GO:0045088 regulation of innate immune response
GO:0032727 positive regulation of interferon-alpha production
GO:0032481 positive regulation of type I interferon production
GO:0045071 negative regulation of viral genome replication
GO:0060337 type I interferon signaling pathway
GO:0032728 positive regulation of interferon-beta production
GO:0042832 defense response to protozoan
GO:0070269 pyroptosis
GO:0045824 negative regulation of innate immune response
GO:0008219 cell death
GO:1901223 negative regulation of NIK/NF-kappaB signaling
GO:0070534 protein K63-linked ubiquitination
GO:0032735 positive regulation of interleukin-12 production
GO:0032731 positive regulation of interleukin-1 beta production
GO:0001817 regulation of cytokine production
GO:0002376 immune system process
GO:0032729 positive regulation of interferon-gamma production
GO:0009615 response to virus
GO:0051607 defense response to virus
GO:0032088 negative regulation of NF-kappaB transcription factor activity
GO:0033209 tumor necrosis factor-mediated signaling pathway
GO:1901224 positive regulation of NIK/NF-kappaB signaling
GO:0050727 regulation of inflammatory response
GO:0007249 I-kappaB kinase/NF-kappaB signaling
GO:0006952 defense response
GO:0007160 cell-matrix adhesion
GO:0043123 positive regulation of I-kappaB kinase/NF-kappaB signaling
GO:0006919 activation of cysteine-type endopeptidase activity involved in apoptotic process
GO:0098586 cellular response to virus
GO:0046330 positive regulation of JNK cascade
GO:0050729 positive regulation of inflammatory response
GO:0050852 T cell receptor signaling pathway
GO:0051092 positive regulation of NF-kappaB transcription factor activity
GO:0045087 innate immune response
GO:0006954 inflammatory response
GO:0071222 cellular response to lipopolysaccharide
GO:0070374 positive regulation of ERK1 and ERK2 cascade
GO:0042981 regulation of apoptotic process
GO:0006915 apoptotic process
GO:0006511 ubiquitin-dependent protein catabolic process
GO:0006955 immune response
GO:0016567 protein ubiquitination
GO:0007165 signal transduction
GO biological processes
5
10
15
Fold enrichment
-log10(FDR)
16
12
8
4
0
Number of genes
10
20
30
40
50

Additional file 9: Gene ontology analysis of DEGs upon IFN treatment compared to non-stimulated condition in the WT cell line (A) and in the *viperin*$^{-/-}$ cell line (B).

GO terms have been filtered to show results with a Benjamini statistical score <0.05. The size of the dot represents the number of genes involved within each biological process and colors represent -log10 (False Discovery Rate).
