# Supplementary material for: Unexpected regulatory functions of cyprinid Viperin on inflammation and metabolism
Source: BMC Genomics. 2024 Jun 29;25:650. doi: 10.1186/s12864-024-10566-x (PMC11218377; doi:10.1186/s12864-024-10566-x)
Supplement: Supplementary file 10 — Additional file 10. KEGG pathway analysis of the DEGs upon IFN treatment compared to the control in the WT and in the viperin-/- cell lines. KEGG pathway terms have been filtered to show results with a Benjamini statistical score <0.05. [file 12864_2024_10566_MOESM10_ESM.pdf]

Ctrl vs. IFN WT
Upregulated genes (n = 165)

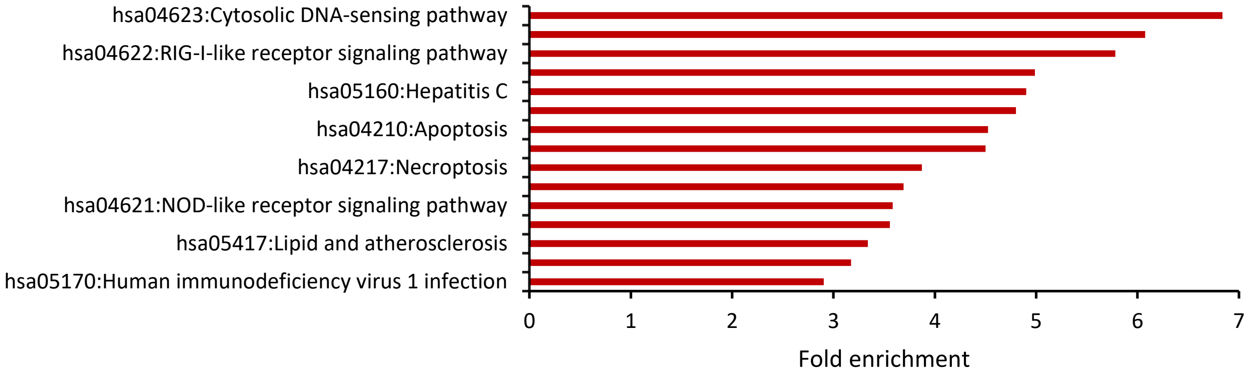

Ctrl vs. IFN KO
Upregulated genes (n = 233)

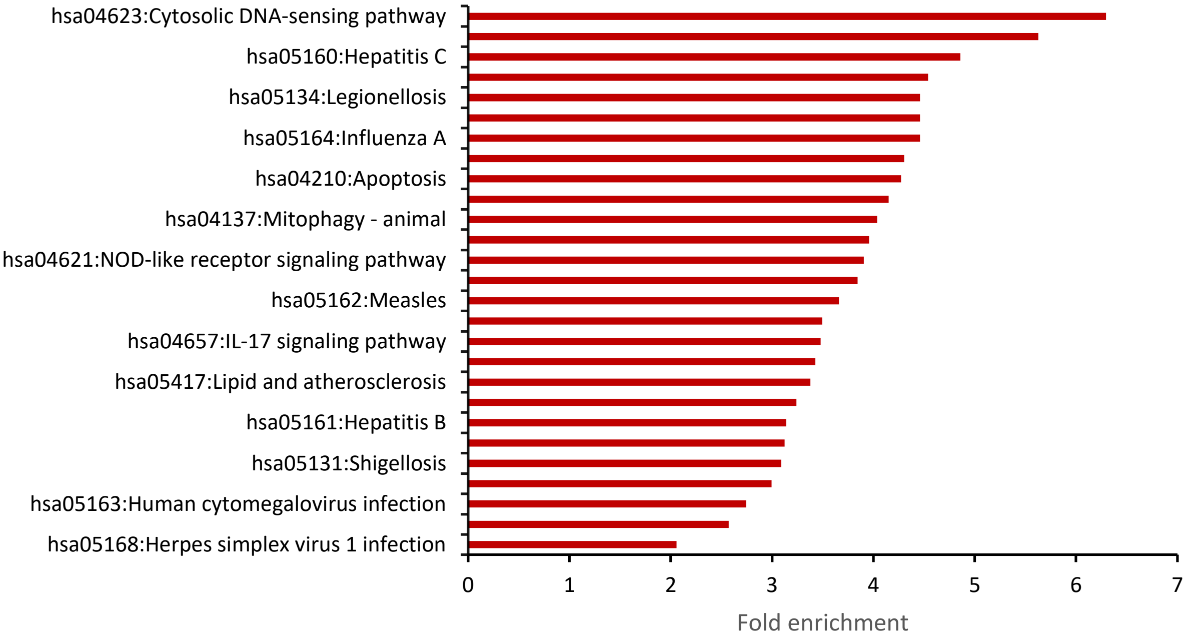

Additional file 10: KEGG pathway analysis of the DEGs upon IFN treatment compared to the control in the WT and in the *viperin*$^{-/-}$ cell lines.

KEGG pathway terms have been filtered to show results with a Benjamini statistical score <0.05.
