# Supplementary material for: Unexpected regulatory functions of cyprinid Viperin on inflammation and metabolism
Source: BMC Genomics. 2024 Jun 29;25:650. doi: 10.1186/s12864-024-10566-x (PMC11218377; doi:10.1186/s12864-024-10566-x)
Supplement: Supplementary file 11 — Additional file 11. Venn diagram showing DEGs in the viperin-/- cell line compared to the WT cell line following type I IFN treatment and previously identified IFNϕ1 modulated genes in zebrafish larvae. The list of IFNφ1 modulated genes comes from Levraud et al., 2019 (56). For comparison purposes, the the zebrafish best Blast hit corresponding to each DEG in the list (considered as the zebrafish ortholog) was used, explaining why some genes are found in both UP and DOWN categories. [file 12864_2024_10566_MOESM11_ESM.pdf]

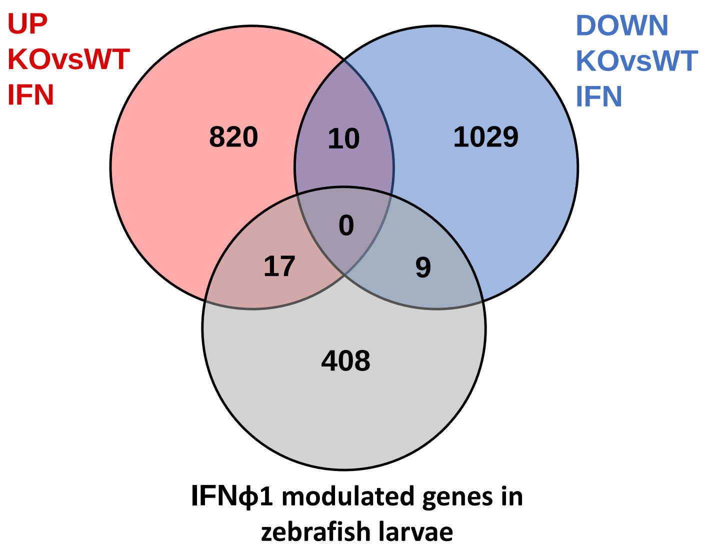

Additional file 11: Venn diagram showing DEGs in the *viperin*$^{-/-}$ cell line compared to the WT cell line following type I IFN treatment and previously identified IFNϕ1 modulated genes in zebrafish larvae.

The list of IFNφ1 modulated genes comes from Levraud et al., 2019 (56). For comparison purposes, the the zebrafish best Blast hit corresponding to each DEG in the list (considered as the zebrafish ortholog) was used, explaining why some genes are found in both UP and DOWN categories.
