# Supplementary material for: Unexpected regulatory functions of cyprinid Viperin on inflammation and metabolism
Source: BMC Genomics. 2024 Jun 29;25:650. doi: 10.1186/s12864-024-10566-x (PMC11218377; doi:10.1186/s12864-024-10566-x)
Supplement: Supplementary file 13 — Additional file 13. KEGG pathway analysis of the DEGs in the viperin-/- cell line compared to the WT cell line at the steady state. KEGG pathway terms have been filtered to show results with a Benjamini statistical score <0.05. Red arrows indicate pathways detailed in Additional file 14. [file 12864_2024_10566_MOESM13_ESM.pdf]

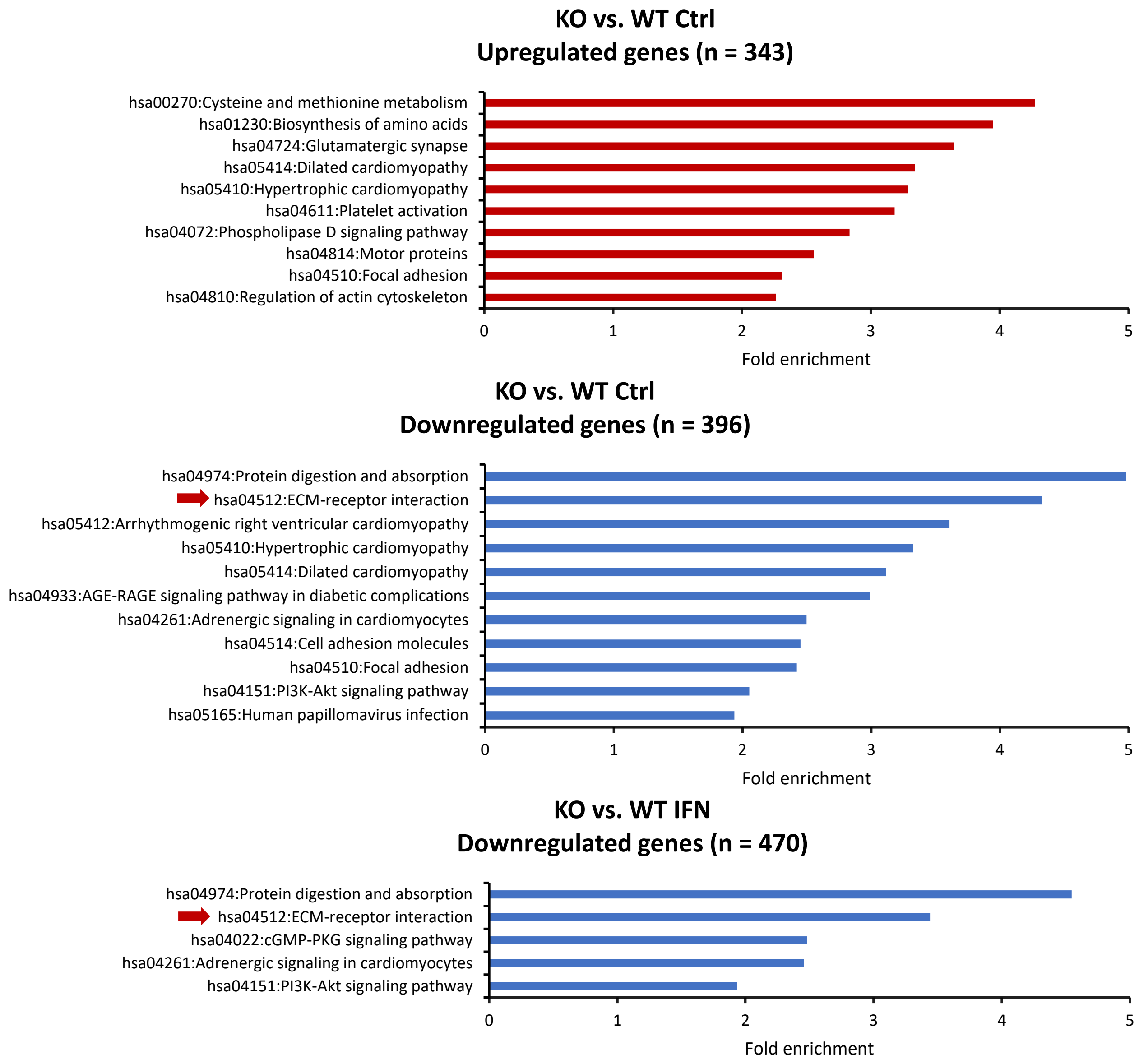


**Additional file 13: KEGG pathway analysis of the DEGs in the *viperin*$^{-/-}$ cell line compared to the WT cell line at the steady state.**

KEGG pathway terms have been filtered to show results with a Benjamini statistical score <0.05. Red arrows indicate pathways detailed in Additional file 14.
