# Supplementary material for: Unexpected regulatory functions of cyprinid Viperin on inflammation and metabolism
Source: BMC Genomics. 2024 Jun 29;25:650. doi: 10.1186/s12864-024-10566-x (PMC11218377; doi:10.1186/s12864-024-10566-x)
Supplement: Supplementary file 14 — Additional file 14. Modulation of ECM-receptor interactions in the viperin-/- cell line compared to the WT cell line at the steady state (upper panel) and upon IFN stimulation (lower panel). DEG datasets were mapped onto the pathway using Pathview. Green and red colors show down- and upregulation, respectively. [file 12864_2024_10566_MOESM14_ESM.pdf]

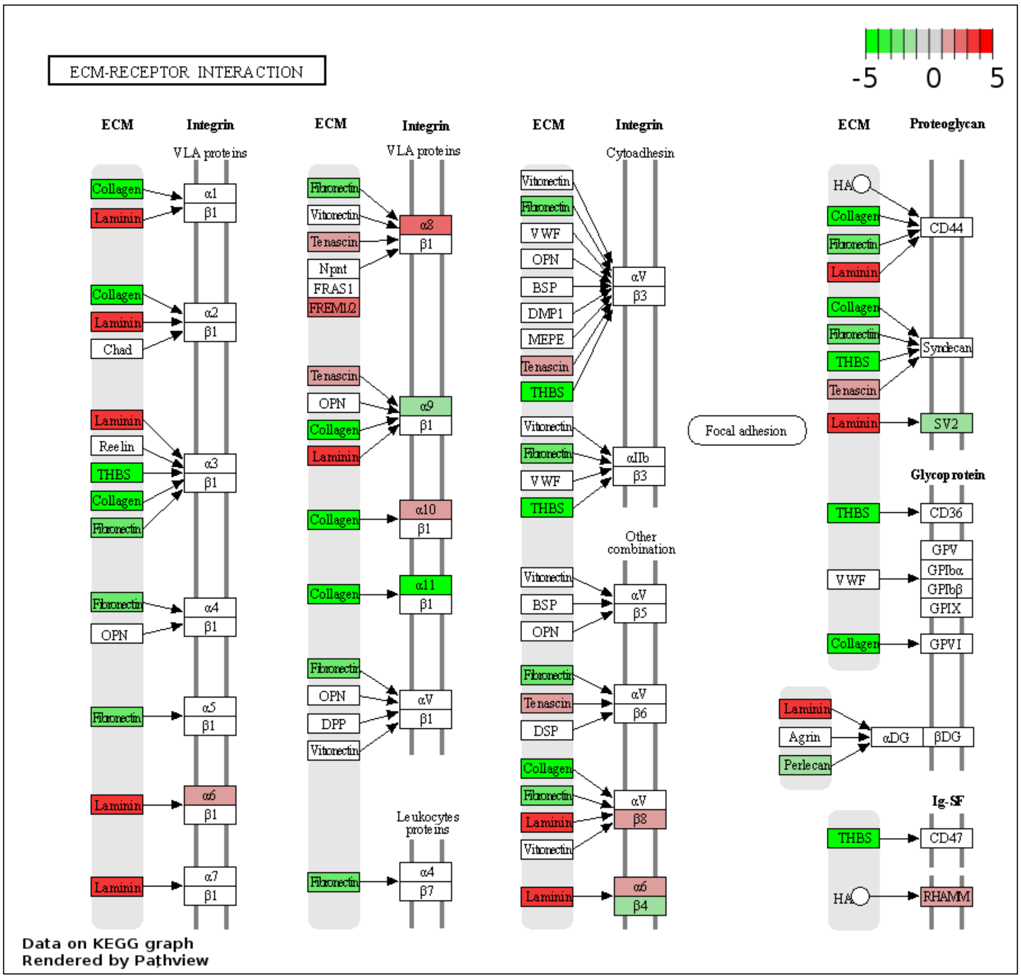

ECM-RECEPTOR INTERACTION
-5
0
5
ECM
Integrin
VLA proteins
Collagen
Laminin
α1
β1
Collagen
Laminin
Chad
α2
β1
Laminin
Reelin
THBS
Collagen
Fibronectin
α3
β1
Fibronectin
OPN
α4
β1
Fibronectin
α5
β1
Laminin
α6
β1
Laminin
α7
β1
ECM
Integrin
VLA proteins
Fibronectin
Vitronectin
Tenascin
Npnt
FRAS1
FREM2
α8
β1
Tenascin
OPN
Collagen
Laminin
α9
β1
Collagen
α10
β1
Collagen
α11
β1
Fibronectin
OPN
DPP
Vitronectin
αV
β1
Leukocytes proteins
Fibronectin
α4
β7
ECM
Integrin
Cytoadhesin
Vitronectin
Fibronectin
VWF
OPN
BSP
DMP1
MEPE
Tenascin
THBS
αV
β3
Vitronectin
Fibronectin
VWF
THBS
αIIb
β3
Other combination
Vitronectin
BSP
OPN
αV
β5
Fibronectin
Tenascin
DSP
αV
β6
Collagen
Fibronectin
Laminin
Vitronectin
αV
β8
Laminin
α6
β4
Focal adhesion
ECM
Proteoglycan
HA
Collagen
Fibronectin
Laminin
CD44
Collagen
Fibronectin
THBS
Tenascin
Syndecan
Laminin
SV2
Glycoprotein
THBS
CD36
VWF
GPV
GPIbα
GPIbβ
GPIX
Collagen
GPVI
Laminin
Agrin
Perlecan
αDG
βDG
Ig-SF
THBS
CD47
HA
RHAMM
Data on KEGG graph
Rendered by Pathview

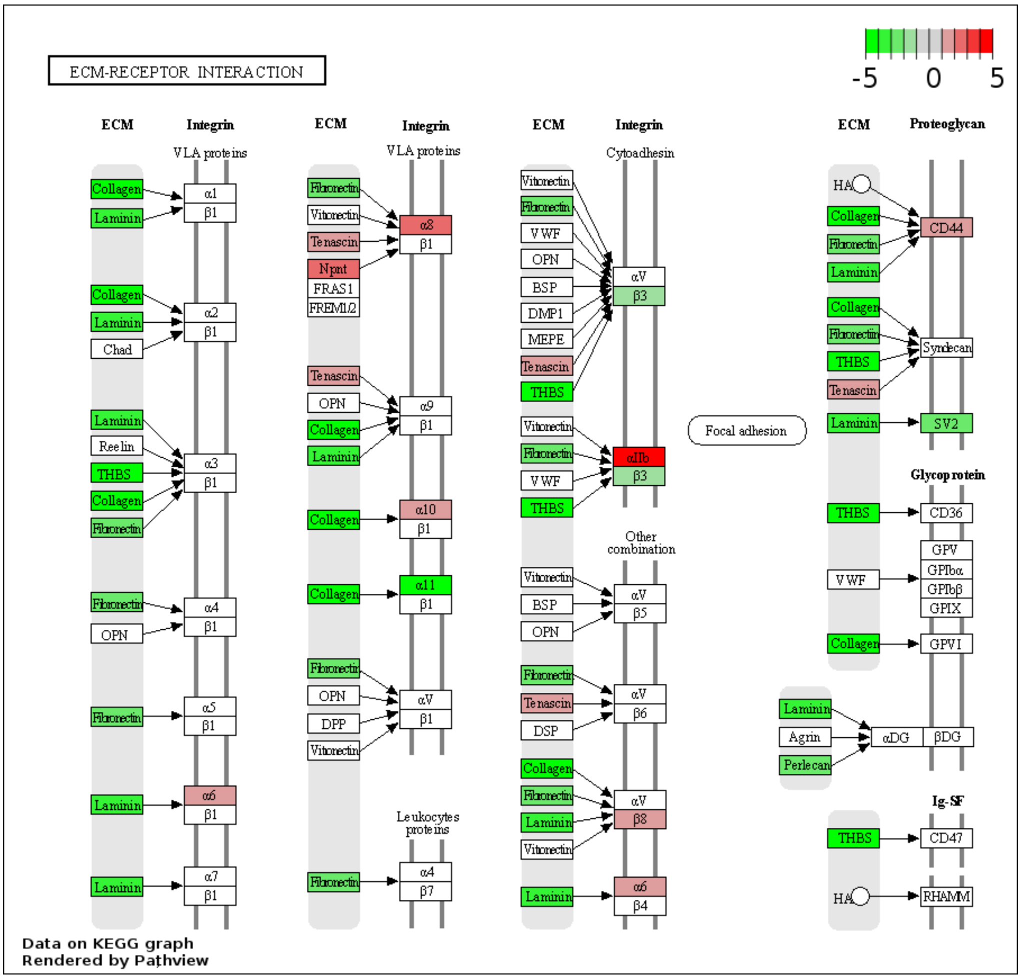

ECM-RECEPTOR INTERACTION
-5
0
5
ECM
Integrin
VLA proteins
Collagen
Laminin
α1
β1
Collagen
Laminin
Chad
α2
β1
Laminin
Reelin
THBS
Collagen
Fibronectin
α3
β1
Fibronectin
OPN
α4
β1
Fibronectin
α5
β1
Laminin
α6
β1
Laminin
α7
β1
ECM
Integrin
VLA proteins
Fibronectin
Vitronectin
Tenascin
Npnt
FRAS1
FREM2
α8
β1
Tenascin
OPN
Collagen
Laminin
α9
β1
Collagen
α10
β1
Collagen
α11
β1
Fibronectin
OPN
DPP
Vitronectin
αV
β1
Leukocytes proteins
Fibronectin
α4
β7
ECM
Integrin
Cytoadhesin
Vitronectin
Fibronectin
VWF
OPN
BSP
DMP1
MEPE
Tenascin
THBS
αV
β3
Vitronectin
Fibronectin
VWF
THBS
αIIb
β3
Other combination
Vitronectin
BSP
OPN
αV
β5
Fibronectin
Tenascin
DSP
αV
β6
Collagen
Fibronectin
Laminin
Vitronectin
αV
β8
Laminin
α6
β4
Focal adhesion
ECM
Proteoglycan
HA
Collagen
Fibronectin
Laminin
CD44
Collagen
Fibronectin
THBS
Tenascin
Syndecan
Laminin
SV2
Glycoprotein
THBS
CD36
VWF
GPV
GPIbα
GPIbβ
GPIX
Collagen
GPVI
Laminin
Agrin
Perlecan
αDG
βDG
Ig-SF
THBS
CD47
HA
RHAMM
Data on KEGG graph
Rendered by Pathview

Additional file 14: Modulation of ECM-receptor interactions in the *viperin*$^{-/-}$ cell line compared to the WT cell line at the steady state (upper panel) and upon IFN stimulation (lower panel).

DEG datasets were mapped onto the pathway using Pathview. Green and red colors show down- and upregulation, respectively.
