# Supplementary material for: Unexpected regulatory functions of cyprinid Viperin on inflammation and metabolism
Source: BMC Genomics. 2024 Jun 29;25:650. doi: 10.1186/s12864-024-10566-x (PMC11218377; doi:10.1186/s12864-024-10566-x)
Supplement: Supplementary file 15 — Additional file 15. Genotyping results from EPC-EC-Vip-C7 and EPC-EC-Vip-C11 subclones. Indels at sgRNA-Vip2 cut site were analyzed using forward sequences with Synthego ICE analysis tool v3. Indels at sgRNA-Vip1 cutsite were manually analyzed using reverse sequences, as ICE was unable to perform the analysis due to the too short reading window around this cutsite. ICE KO-score indicates the proportion of indels leading to a frameshift; R² indicates how well the proposed distribution fits the sequence of the edited sample. [file 12864_2024_10566_MOESM15_ESM.pdf]

Additional file 15: Genotyping results from EPC-EC-Vip-C7 and EPC-EC-Vip-C11 subclones.

Parental cell line	Subclones		sgRNA-Vip2 TGGAGTGGTCACCTGTGCGC					sgRNA-Vip1 CGAACGAGGTCTTCGCAGTG		
			Sequence	Analysis type	KO-Score	R^2	Indel	Sequence	Analysis type	Indel[a]
EPC-EC-Vip-C7	**1**	**EPC-EC-Vip-C7-sub2**	Vip-gen-F	ICE	100	0.98	{-1: 71% ; -2: 29%}	Vip-gen-R	Manual	+1/WT
	**2**	**EPC-EC-Vip-C7-sub3**	Vip-gen-F	ICE	100	0.98	{-1: 70% ; -2: 30%}	Vip-gen-R	Manual	+1/WT
	**3**	**EPC-EC-Vip-C7-sub4**	Vip-gen-F	ICE	99	0.98	{-1: 69% ; -2: 28%}	Vip-gen-R	Manual	+1/WT
	**4**	**EPC-EC-Vip-C7-sub5**	Vip-gen-F	ICE	100	0.98	{-1: 71% ; -2: 29%}	Vip-gen-R	Manual	+1/WT
	**5**	**EPC-EC-Vip-C7-sub6**	Vip-gen-F	ICE	100	0.97	{-1: 67% ; -2: 21%}	Vip-gen-R	Manual	+1/WT
	**6**	**EPC-EC-Vip-C7-sub8**	Vip-gen-F	ICE	100	0.98	{-1: 71% ; -2: 29%}	Vip-gen-R	Manual	+1/WT
	**7**	**EPC-EC-Vip-C7-sub9**	Vip-gen-F	ICE	100	0.98	{-1: 70% ; -2: 29%}	Vip-gen-R	Manual	+1/WT
	**8**	**EPC-EC-Vip-C7-sub10**	Vip-gen-F	ICE	100	0.98	{-1: 51% ; -2: 48%}	Vip-gen-R	Manual	ND
	**9**	**EPC-EC-Vip-C7-sub11**	Vip-gen-F	ICE	99	0.98	{-1: 69% ; -2: 30%}	Vip-gen-R	Manual	+1/WT
	**10**	**EPC-EC-Vip-C7-sub12**	Vip-gen-F	ICE	100	0.98	{-1: 70% ; -2: 28%}	Vip-gen-R	Manual	+1/WT

EPC-EC-Vip-C11	**1**	**EPC-EC-Vip-C11-sub1**	Vip-gen-F	ICE	100	0.98	{-1: 71% ; -2: 28%}	Vip-gen-R	Manual	WT/+1
	**2**	**EPC-EC-Vip-C11-sub2**	Vip-gen-F	ICE	100	0.98	{-1: 72% ; -2: 26%}	Vip-gen-R	Manual	WT/+1
	**3**	**EPC-EC-Vip-C11-sub4**	Vip-gen-F	ICE	100	0.98	{-1: 70% ; -2: 30%}	Vip-gen-R	Manual	WT/+1
	**4**	**EPC-EC-Vip-C11-sub5**	Vip-gen-F	ICE	100	0.98	{-1: 71% ; -2: 29%}	Vip-gen-R	Manual	WT/+1
	**5**	**EPC-EC-Vip-C11-sub7**	Vip-gen-F	ICE	100	0.98	{-1: 73% ; -2: 27%}	Vip-gen-R	Manual	WT/+1
	**6**	**EPC-EC-Vip-C11-sub9**	Vip-gen-F	ICE	100	0.98	{-1: 71% ; -2: 29%}	Vip-gen-R	Manual	WT/+1
	**7**	**EPC-EC-Vip-C11-sub10**	Vip-gen-F	ICE	100	0.98	{-1: 69% ; -2: 28%}	Vip-gen-R	Manual	WT/+1

Indels at sgRNA-Vip2 cut site were analyzed using forward sequences with Synthego ICE analysis tool v3. Indels at sgRNA-Vip1 cutsite were manually analyzed using reverse sequences, as ICE was unable to perform the analysis due to the too short reading window around this cutsite. ICE KO-score indicates the proportion of indels leading to a frameshift; R^2 indicates how well the proposed distribution fits the sequence of the edited sample.

[a] Percentage values could not be inferred but the order indicates which sequence is predominant in the chromatograms (e.g. in EPC-EC-Vip-C7-sub2, the sequence presenting a 1-nt insertion is predominant over the WT sequence)
