# Supplementary material for: Unexpected regulatory functions of cyprinid Viperin on inflammation and metabolism
Source: BMC Genomics. 2024 Jun 29;25:650. doi: 10.1186/s12864-024-10566-x (PMC11218377; doi:10.1186/s12864-024-10566-x)
Supplement: Supplementary file 16 — Additional file 16. Comparison of expression pattern of genes (n=925) located on the scaffold NW_0241210099.1 in EPC-EC-Vip-C7 cells compared to EPC-EC cells at the steady state (Ctrl) and following IFN treatment (IFN). No increase in “gene expression” was detected. ns, non-significant, one sample t-test. [file 12864_2024_10566_MOESM16_ESM.pdf]

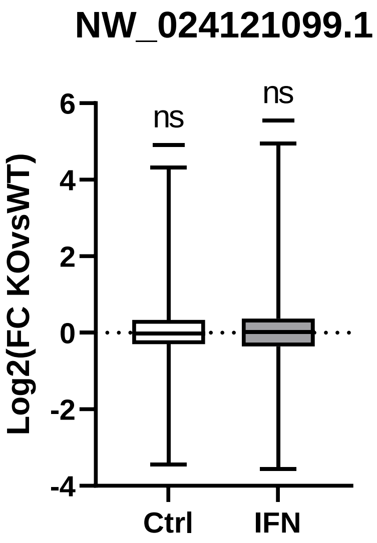

Additional file 16: Comparison of expression pattern of genes (n=925) located on the scaffold NW_0241210099.1 in EPC-EC-Vip-C7 cells compared to EPC-EC cells at the steady state (Ctrl) and following IFN treatment (IFN).

No increase in “gene expression” was detected. ns, non-significant, one sample t-test.
